# Opposite differential risks for autism and schizophrenia based on maternal age, paternal age, and parental age differences

**DOI:** 10.1093/emph/eow023

**Published:** 2016-08-16

**Authors:** Sean G. Byars, Jacobus J. Boomsma

**Affiliations:** 1 Centre for Social Evolution, Department of Biology, University of Copenhagen, Copenhagen, Denmark; 2 Department of Pathology, The University of Melbourne, Parkville, VIC 3010, Australia; 3 Present address: Centre for Systems Genomics, School of BioSciences, The University of Melbourne, Parkville, VIC 3010, Australia.

**Keywords:** mental disease, risk assessment, parent-offspring conflict, genomic imprinting, life history theory, parental-conflict

## Abstract

**Background and objectives:** Effects of maternal and paternal age on offspring autism and schizophrenia risks have been studied for over three decades, but inconsistent risks have often been found, precluding well-informed speculation on why these age-related risks might exist.

**Methodology:** To help clarify this situation we analysed a massive single population sample from Denmark including the full spectrum of autistic and schizophrenic disorders (eliminating between-study confounding), used up to 30 follow-up years, controlled for over 20 potentially confounding factors and interpret the ultimate causation of the observed risk patterns using generally accepted principles of parent-offspring conflict and life-history theory.

**Results:** We evaluated the effects of paternal age, maternal age and parental age difference on offspring mental disorders and found consistently similar risk patterns for related disorders and markedly different patterns between autistic and schizophrenic disorders. Older fathers and mothers both conferred increased risk for autistic but not schizophrenic disorders, but autism risk was reduced in younger parents and offspring of younger mothers had increased risk for many schizophrenic disorders. Risk for most disorders also increased when parents were more dissimilarly aged. Monotonically increasing autism risk is consistent with mutation accumulation as fathers’ age, but this explanation is invalid for schizophrenic disorders, which were not related to paternal age and were negatively correlated with maternal age.

**Conclusions and implications:** We propose that the observed maternally induced risk patterns ultimately reflect a shifting ancestral life-history trade-off between current and future reproduction, mediated by an initially high but subsequently decreasing tendency to constrain foetal provisioning as women proceed from first to final pregnancy.

## Teaser

Parents' age affects autism and schizophrenia risk, but large-scale single-population studies covering the entire mental disease spectrum are lacking. For a nation-wide Danish data set, we show that older fathers increase risks for autism but not schizophrenia, and that mothers transition from imposing increased schizophrenia to increased autism risk as they age.

## INTRODUCTION

Since the first single-disorder surveys that discovered an association between risk of autism or schizophrenia and advancing maternal or paternal age over 30 years ago [1, 2], there have been numerous studies that have attempted to confirm this pattern, including several very recent ones [3–6]. Despite this substantial effort, risk patterns for the same disorders have been found to be highly variable and results often remain incomparable due to substantial differences in study design (Supplementary Table S1). For example, McGrath *et al.* [6] found that risk of childhood autism was significantly higher in offspring born to younger mothers, Parner *et al.* [7] and Shelton *et al.* [8] found risk significantly higher in older mothers, while other studies show U-shaped risk patterns [3, 5]. For paternal age, McGrath *et al.* [6] and others [3, 7] found that autism (or autism-spectrum disorder) risk was significantly higher for offspring born to older fathers, while Lampi *et al.* [5] and Lundstrom *et al.* [9] found significant U-shaped risk patterns.

Results of schizophrenia research show similar variability (Supplementary Table S1). For maternal age, studies show younger [6, 10], older [4] or both age extremes [11] contributing risk, and studies of paternal age implicate older [6, 10] or both younger and older [11–13] fathers as risk factors. It is likely that these between-study differences reflect artifactual variation in study design, for example due to massive differences in sample size, statistical power, follow-up time, covariates adjusted for, and time periods over which populations were considered. For example, Rasmussen [14] used 22–29 years of follow-up from birth while McGrath *et al.* [6] used 5–19 years, which will only capture a small percentage of total schizophrenia incidence because diagnoses typically occur between adolescence (ca. 20 years) and middle age with a second peak for women between ca. 45–80 years [15]. Also sample sizes have varied widely, ranging from a few hundred [10, 16] to well over a million [7, 8] (see Supplementary Table S1 for details). It therefore remains critical to clarify what the true effects of parental age on autism and schizophrenia risk are to enable better understanding of their aetiology, putative evolutionary explanation, and relevance for clinical risk management.

Recent meta-analyses have attempted to reduce outcome variability by combining coefficients from different studies in order to distil overall risk patterns, including paternal age and autism [17], or schizophrenia [12], and maternal age and autism [18]. However, this approach is limited by the quality or variation of input coefficients that may already be biased, for example by differences in detection capabilities and diagnostic criteria by country and cultural differences in the ages parents typically have babies. Despite inconsistent risk patterns for both autism and schizophrenia, the popular interpretation that *de novo* mutations in sperm are likely responsible for the increased risk seen in offspring of older fathers has garnered considerable mass media attention (e.g. [19]). While mutations accumulate linearly in sperm with age, this correlation does not confirm a direct genotype–phenotype link between these mutations and autism and schizophrenia. This inference is also premature given that some studies show the youngest fathers may have offspring with higher psychiatric disorder risks [12] and that the effects of both parents being older (vs only one being older) may not be cumulative [7]. A recent study also showed that maternal germline mutations do accumulate albeit at a lower rate and non-linearly [20], and it has also been argued that risks are primarily determined by parental age at first reproduction [21] and parity [4] rather than by parental age as such. These complexities imply that large-scale longitudinal studies comparing psychiatric disorder risks within a single population are required to partial out important confounding effects.

The need for large-scale rigorous studies is also evident from most studies only accounting for three to seven covariates (Supplementary Table S1) such as parity, gender, family history and some socioeconomic factors. This leaves unexplored age-correlated (and potentially causal) effects such as maternal hypertension and diabetes, pregnancy complications such as previous spontaneous or induced abortions, maternal bleeding during pregnancy, foetal oxygen deprivation, gestational diabetes, pregnancy oedema, and gestational hypertension. Also any effects of age-difference between partners can only be handled in large data sets. For example, in studies where older fathers and mothers both independently contribute risk to autism [7, 8] it is valid to ask whether risk is ameliorated when one parent is much younger, but there are only a few studies that have examined this, and only for autism [7, 8, 22]. Durkin *et al.* [22] found risk tended to be highest when both parents were older compared with when one or both were younger, but Parner *et al.* [7] found no compounding effect and Shelton *et al.* [8] found that risks were highest when older fathers (>40 years) reproduced with younger mothers (<30 years).

While proximate explanations for mental disorders (e.g. mutation accumulation in sperm, obstetric complications in mothers) seem straightforward to conceptualize, we are still a long way from understanding why variation in parental age should affect offspring psychiatric disorder risk, i.e. grasping how any such effects may have been maintained in human populations that respond to natural selection. Over recent years, several branches of evolutionary theory have yielded useful paradigms that can fruitfully be applied to such ultimate questions of human reproductive and mental health.

First, parent-offspring conflict (POC) theory developed by Trivers [23] predicts that maternal life-time reproductive success is normally best served by provisioning current and future offspring equally, while each offspring has been under selection to secure more resources to self than to potential future siblings. This well-known conflict is driven by focal offspring being 100% related to self and 50% to future siblings when parents remain monogamous for life. Thus, offspring resource interests that diverge from the mother’s optimum are naturally driven to do so by relatedness asymmetries [24] because offspring inherit only half of their genome from the mother. When there is multiple paternity so future offspring will have some likelihood of being half-siblings, paternally inherited genes in offspring should favour even higher maternal resource acquisition relative to maternally inherited genes in offspring.

The related Parental-Conflict (PC) theory subsequently developed by Haig [25] addresses maternal-foetal conflict over resources during pregnancy, mediated by ‘matrigenic’ and ‘patrigenic’ influences (terms coined by Queller [26]) on placental and foetal physiology. These effects emanate from differential expression of genes with parent-of-origin genomic imprints and/or copy-number variation (Haig [27] refers to these effects as ‘madumnal’ and ‘padumnal’ genes). Such patrigenic effects achieve higher fitness when they improve offspring growth and survival beyond the maternal optimum and will tend to be compensated by matrigenic effects that are growth restricting [28]. PC theory was recently used to propose the Imprinted Brain (IB) theory [29] hypothesizing that these matrigenic/patrigenic conflicts have extended postnatal effects when they manipulate parental and particularly maternal investment until weaning. Here, genes with parent-of-origin effects are expected to be expressed in the offspring brain and to induce higher or lower demands for resources. Patrigenic and matrigenic effects normally balance to give average (normal) cognition, but deviations are expected to increase risks of mental disorders in specific directions such that matrigenic bias gives higher schizophrenia and lower autism risks, whereas patrigenic bias gives higher autism and lower schizophrenia risks. There is some correlational genetic [29, 30] and more extensive phenotypic support [31–34] for IB theory.

Second, Life History (LH) theory characterizes trade-offs between current and future reproduction during the typical life course of parents–particularly the mother [35, 36], and provides a more general cost-benefit context for POC, PC and IB theory [27, 37]. LH theory predicts that the intensity of conflict should decrease with increasing maternal age because her reproductive value–the likelihood of raising any future offspring towards independence after a focal pregnancy–declines rather steeply with age [23]. We thus expect higher than average restraints for mothers to invest in offspring provisioning during early pregnancies graduating into above average provisioning in later pregnancies as the likelihood of future offspring declines towards zero when she approaches menopause [e.g. 36, 38, 39]. This predictable gradient in the life of every woman is expected to have selected for tighter control of patrigenic pressure during early pregnancies and their associated postnatal offspring behaviour and for a gradual relaxation of this control in later pregnancies, giving more leeway to patrigenically mediated interests. Thus, combining insights from IB [40] and LH theories allows the prediction that the balance between infant risk profiles for autism and schizophrenia shifts from schizophrenia-biased to autism-biased across the time window that women typically reproduce, and that these effects could be reinforced or ameliorated by paternal age.

No study has formally conceptualized and tested the validity of these inferences, which follow directly from the classic distinction that female reproductive success is normally limited by access to resources whereas male reproductive success is limited by access to females [41]. Moreover, no previous study has simultaneously tested and compared parental age effects on autism- and schizophrenia-spectrum conditions in the same population. As we had access to 30 years of national public health data in Denmark, we designed the present study to rigorously assess the effects of both parents’ age, their age difference, and more than 20 covariates broadly across all autistic and schizophrenic disorders. Our objectives were to interpret the results in both proximate (mechanistic) and ultimate (evolutionary) terms to further general understanding of what risks of specific mental disorders (co)vary with parental age, or not, and whether risks that can be unambiguously documented are consistent with explanations from evolutionary theory, or not.

## MATERIAL AND METHODS

We compared risk patterns between autistic, schizophrenic and related disorders based on deviations from average maternal, paternal and parental age differences. We used Cox regression (proportional hazards) to model the risk of psychiatric disorders as a function of parental age and all available covariates to account for environmental and familial effects on these disorders. To correct for multiple testing (i.e. regressions run for each of the ten mental disorder groups described below), all *P*-values were Bonferroni corrected (*±* = 0.05/10 = 0.005).

### Parental age variables categorically defined

Maternal age, paternal age and parental age difference variables were classified categorically into seven groups (including a mean reference group). Size of age breaks for maternal, paternal or age difference groups were each approximated using standard deviations from their means, i.e. groups 3–4 approximate ages up to 1 standard deviation of the mean for each variable. Each group was coded as a binary variable that represented whether ages fell below (groups 1–3) or above (groups 4–6) the mean (Supplementary Table S2): group 1 (fathers 16–20 years, mothers 15–21 years; mothers 8–14 years older than fathers); group 2 (fathers 21–25 years, mothers 22–24 years; mothers 4–7 years older); group 3 (fathers 26–30 years, mothers 25–28 years; mothers 1–3 years older); central group (fathers 31–34 years, mothers 29–31 years; fathers 0–6 years older than mothers); group 4 (fathers 35–39 years, mothers 32–34 years; fathers 7–10 years older); group 5 (fathers 40–44 years, mothers 35–38 years; fathers 11–15 years older); group 6 (fathers 45–60 years, mothers 39–46 years; fathers 16–27 years older).

### Study population

We identified all singleton live births (*n* = 1 787 447) between January 1978 and January 2009 in the Danish Fertility Database, which contains identification of parents and other birth-related information. We used unique personal identification numbers (de-identified from central-person register numbers) to link individual’s information between different registries including the Danish National Patient Registry holding nationwide hospital admission data since 1977, the Danish Psychiatric Central Register with diagnoses for all psychiatric inpatient admissions since 1969, the Danish Civil Registration System and the Danish Cause of Death Registry containing date of death, migration, and socioeconomic status. The sample of singleton births was reduced from 1 787 447 to 1 740 269 due to missing values for either parents’ age (*n* = 29 538), parity (*n* = 69), APGAR5 score (*n* = 10 699; defined below); extreme birth weight values <1850 and >5400 g (*n* = 1624); extreme paternal (<16 and >60, *n* = 852) and maternal age (<15 and >46, *n* = 181) values; and very short gestation (<30 weeks, *n* = 4215). The final sample included 1 646 092 offspring that were never diagnosed with a mental disorder during the study period while 94 177 (5.41%) were diagnosed within one of the autistic or schizophrenic disorder groups defined below (Supplementary Table S2).

### Defining autistic and schizophrenic disorder groups

As per a previous study [31], we included a range of narrow to broad autistic and schizophrenic disorder groups (ICD codes, Supplementary Table S3). Including a range of related disorders helps compare and validate the risk patterns found. Broader groups have the statistical advantage of larger case sample sizes while narrower groups help improve resolution on specific diagnoses more commonly given in a clinical setting. Narrow to broad autistic (and related) disorder groups included: (i) infantile autism; (ii) infantile and atypical autism; (iii) autism-spectrum disorders that covered autism (infantile and atypical), Asperger’s syndrome, pervasive developmental disorder not otherwise specified; (iv) disorders of psychological development; (v) behavioural and emotional disorders with onset in childhood and adolescence. Narrow to broad schizophrenic (and related) disorder groups included: (i) bipolar disorder; (ii) major depression; (iii) schizophrenia; (iv) schizophrenia-spectrum disorders that included schizophrenia, bipolar disorder, and major depression; (v) schizophrenia, schizotypal and delusional disorders. For details on sources and validation of mental illness, see Supplementary Methods and Results.

### Covariates included in the analyses

Covariates came from diagnoses and measures obtained from the Danish birth, psychiatric, person and household registries across 1978–2009. These were included as predictor variables in Cox regressions and helped to partial out potential confounding effects on mental disorder variation. They capture important heritable, pregnancy, birth and socioeconomic effects known to modify (mental) disorder risk including: binary variables for maternal pre-existing conditions (see Supplementary Table S4 for ICD codes) including pre-existing hypertension (primary or secondary hypertension, hypertensive heart or renal disease), pre-existing diabetes (i.e. type-I or type-II, malnutrition-related, other or unspecified), previous spontaneous or induced abortions; maternal pregnancy-related or induced (Supplementary Table S4) variables including gestation length (in weeks) and binary variables for presence of gestational diabetes, gestational hypertension (i.e. pregnancy-induced hypertension, preeclampsia, eclampsia), bleeding (i.e. haemorrhage, placenta praevia), foetal oxygen deprivation (i.e. hypoxia, asphyxia), pregnancy oedema; parental variables including a binary marker for whether either parent had ever been given a psychiatric diagnosis within the same disorder group as the child to account for familial transmission, total number of years of education summed across both parents and average income (in Dkr) across the study period summed across both parents; birth-related variables including birth weight (in grams), birth season (calendar month, 1–12), birth year (linear variable with 3-year cohorts between 1978 and 2008 accounting for changes in diagnostic criteria over time), and the APGAR5 (Appearance, Pulse, Grimace, Activity, Respiration) score of 1–10 (maximally 2 points for each category) given to babies shortly after birth ranging from poor to excellent health; other child-related variables including offspring sex (0 = male, 1 = female), nationality (0 = Danish national, 1 = immigrant), demographic parity, region within Denmark (Hovedstaden = Copenhagen Area, Sjælland, Syddanmark, Midtjylland and Nordjylland) a child had resided for the longest period to account for possible regional differences in diagnoses. We also ran analyses including total years of education and average income for mothers and fathers separately, but this made no difference to the main parental age predictors, quite likely because education in Denmark is free for all residents.

## RESULTS

### Advanced parental age at birth

**Figure 1.**
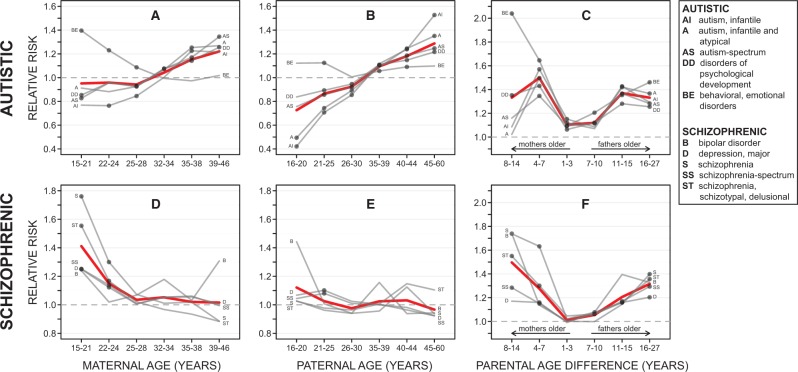
Risk of offspring psychiatric disorders by parental age. Plots are divided by autistic (**A–C**) versus schizophrenic (**D–F**) disorders and by maternal age (A, D), paternal age (B, E) and parental age difference (C, F) at birth. Dashed horizontal lines (RR = 1.0) indicate zero risk. For parental age difference plots, groups left of centre represent mothers 1–3, 4–7 or 8–14 years older than their reproductive partners at childbirth. Groups to the right are fathers 7–10, 11–15 or 16–27 years older than their reproductive partners. Dark-grey dots mark risk *P*-values <0.05. All *P*-values were Bonferroni-corrected before further interpretation. Key provides full autistic and schizophrenic disorder group names for abbreviations in plots

Above-average paternal and maternal ages were consistently associated with increased risk of most autistic (and related) disorders in offspring (Fig. 1A and B). This effect was magnified in offspring of very old fathers and mothers as risk continued to increase in age groups further from the reference (central) group. For example, across the three older paternal age groups (groups 4–6 or ages 35–60) relative risks for all five autistic disorders in offspring were elevated with 12 out of those 15 coefficients significantly increased relative to the reference group (5–52% increased risk, Fig. 1B, Supplementary Tables S5–S9).

Trends for the five autistic disorders were also largely consistent across the three older maternal age groups (groups 4–6 or ages 32–46), with elevated relative risk for 13 out of 15 coefficients (8 of these were significant, 7–34% increased risk, Fig. 1A, Supplementary Tables S5–S9). However, neither advanced maternal nor paternal ages were associated with significantly modified risk of any schizophrenic disorder groups (Fig. 1D and E, Supplementary Tables S10–S14).

### Young parental age at birth

Below average paternal ages (groups 1–3, ages 16–30) appeared to have a protective effect on autistic disorder risk, with relative risk for four of these disorders consistently decreased. Risks continued to decrease the further away from the central group, with some offspring disorders displaying large significant effects (e.g. 14–57% decreased risk for infantile autism, 10–50% decreased risk for autism in offspring of the youngest fathers) (Fig. 1B, Supplementary Tables S5 and S6). This protective effect for autism was also largely consistent in offspring born to younger mothers (groups 1–3, ages 15–28). For example, risk was significantly reduced by 15–23% for infantile autism, and by 7–17% for autism-spectrum disorders (Fig. 1A, Supplementary Tables S5 and S7). While below average maternal and paternal ages were both protective for autism, this effect was more pronounced in younger paternal age groups. The only exception was behavioural and emotional disorders, which showed increased relative risk in below-average paternal and maternal age groups (Fig. 1A and B). This may be due to this disorder being more distantly related to the other autistic-related disorders (see psychiatric diagnostic structure, Supplementary Fig. S1 in Byars *et al.* [31]), i.e. it captures disorders with specific onset in childhood and adolescence, but does not include the core phenotypes of autism.

Patterns of risk for the five schizophrenic disorders in offspring born within the three younger than average maternal age groups (i.e. groups 1–3 or ages 15–28) were consistently increased with all risk ratios above 1.0, and 8 out of those 15 coefficients being significant for schizophrenia-spectrum disorders, schizophrenia, major depression and schizophrenia-schizotypal and delusional disorders (Fig. 1D). However, risk for the five schizophrenic disorders in offspring were largely unaffected across the three younger than average paternal age groups (i.e. groups 1–3 or ages 16–30), with only 2 (of 15) risk ratios marginally significantly increased (i.e. ages 21–25: *P*-value = 0.04 for schizophrenia-spectrum disorders; *P*-value = 0.02 for major depression) (Fig. 1E).

### Parental age difference

As well as investigating age of both parents, the parental age difference variable allowed us to separate dissimilar parental ages at birth, with values on the left side of the distribution corresponding to younger fathers reproducing with older mothers and values on the right related to younger’s mothers reproducing with older fathers (Fig. 1C and F). Overall, autistic and schizophrenic risk in offspring was lowest for more similarly aged parents. Risk for autism was higher towards the distribution edges, with risk on the right side (i.e. groups 4–6, fathers aged 7–27 years older than mothers) consistently more significant than the left (i.e. groups 1–3, mothers aged 1–14 years older than fathers), especially at the distribution extremes (groups 1 and 6, Fig. 1C). Schizophrenia risk was also largely U-shaped, with risk for most disorders increasing as parental age differences increased (Fig. 1F). This was similar on both sides for the five schizophrenic disorders, with 7 out of 15 risk ratios significantly increased in the three age-difference groups on the right (i.e. groups 4–6, fathers aged 7–27 years older than mothers) and 9 out of 15 significantly increased for the three age-difference groups on the left (i.e. groups 1–3, mothers aged 1–14 years older than fathers).

### Risk patterns for covariates

Offspring risk of many psychiatric disorders increased if mothers or fathers had the same disorder, if there were diabetes or hypertension complications during pregnancy, previous abortions, and if offspring were born more recently. Risks were often decreased if babies were born closer to term, had birth weights closer to the mean and higher APGAR5 scores. Our earlier study [31] provides an extensive analysis of birth size effects on risks of autistic and schizophrenic disorders later in life, so we will not report on these differences in any detail here. As we found previously, in our current analyses the risk of autistic disorders was generally higher in sons and the risk of schizophrenic disorders generally higher in daughters. Overall, just over half of the 22 covariates were highly significant (*P* < 0.001) across all 10 analyses (Supplementary Tables S5–S14) highlighting the importance of accounting for these effects. For further discussion of these covariate effects, see Supplementary Methods and Results and our previous study [31]. To quantify potential maternal age-related effects on foetal provisioning, we obtained *post hoc* correlations between maternal age and offspring birth statistics. Most size and health (APGAR5) variables at birth were positively and significantly correlated with maternal age, but the variation directly explained by maternal age was minor given the small Pearson correlation coefficients (Supplementary Table S15).

## DISCUSSION

### Risk patterns for autism–maternal age appears to matter

Our results lend support to many previous studies (see metanalyses, [17, 18]) that have indicated that above-average paternal and maternal ages are independently linked to increased risk of autism in their offspring. This effect has recently been discussed in relation to increasing mutational load in sperm of older fathers [42], a pattern that may correlate with mental disorder prevalence [19], although it is still being debated whether these mutations contribute directly to altered neurodevelopmental phenotypes [4]. Visually, the change in autistic risk across the full paternal age range (Fig. 1B) appeared to be roughly monotonic, consistent with a proximate mechanism such as mutational load that accumulates steadily over time [42].

This type of explanation is harder to maintain for older mothers, although age-related novel mutational events [20, 43, 44], epigenetic changes [18, 45] or other factors might plausibly be involved [18, 46]. Considering that our analyses adjusted for both parents’ ages and that effects from our parental age difference variable also supported that older mothers confer increased risk of autism on their offspring, this effect appears to be important. Supporting this, a recent study showed that the frequency of aneuploidy (which is linked to autism risk [47]) increases considerably in older mothers [48]. A more specific focus on maternal age risk factors is therefore needed, especially considering that most studies have focused on interpreting autism risk from a paternal perspective only.

### No effect of advanced paternal or maternal age on schizophrenia risk

Our results were consistent with two recent studies that also found no effect of advanced parental age on risk of schizophrenia [6, 10]. This contrasts with studies that have found schizophrenia risk in offspring to be associated with above-average paternal [11, 12, 49–55] or maternal age [11, 51, 53]. However, many of these studies had relatively low sample sizes and controlled for few confounding effects (e.g. [49, 56, 57]). Such design limitations are likely to introduce power issues or errors in reliably detecting similarities or differences in risk between age groups. Large differences in key design features and risk trends compared across studies in Supplementary Table S1 indicate that the impression of consistency across studies conveyed by some previous studies is misleading.

When interpreting results across studies, it is also important to note that schizophrenia is inherently more difficult to study than autism given that it is detected much later, from around age 18 onwards. In particular for women, detection may not occur until after 40 years of age [15] implying that many studies with less follow-up time will miss many relevant diagnoses. Indeed, large variation in follow-up time among studies exists (Supplementary Table S1) ranging from 15 to 38 years from birth. In our study, we had up to 30 years of follow-up, which should allow detection of enough cases for reliable risk estimation due to our very large sample sizes, but we are likely still missing a substantial number of late-onset schizophrenia cases in the Danish population.

### Increased schizophrenia risk due to younger maternal ages

Younger mothers conferring schizophrenia risk to their offspring has been found previously although results have often not been clear within or consistent between studies. For example Byrne *et al.* [11] found mothers aged <20 conferred higher schizophrenia risk, although this effect disappeared in adjusted models. Frans *et al.* [51] found no effect of maternal ages <20 on risk of bipolar disorder, Malaspina *et al.* [52] found no effect of maternal ages below 20 on schizophrenia, and Wu *et al.* [10] found increased risk of schizophrenia in offspring of mothers <25 years of age.

More recently and with a large sample size, McGrath *et al.* [6] found that younger mothers aged 12–19 and 20–24 conferred significantly increased risk for schizophrenia, schizophrenia and related disorders, mood disorder, bipolar disorder, similar to the results of our present study. We found increased risk for schizophrenia, schizophrenia-spectrum, major depression and schizophrenia-schizotypal-delusional disorders in younger maternal age groups 15–21 and 22–24 years, but risks did not change monotonically across the entire age range (compared with autism) (Fig. 1D), suggesting that psychosocial, cultural or resource-mediated factors might also be involved. For example, effects of early maternal age at birth on offspring schizophrenia risk have been linked to social and environmental stress factors that may be more common in earlier reproducing families [58].

### Higher risks in offspring of dissimilarly aged parents

Previously, only Croen *et al.* [59] examined the effect of parental age differences in autism-spectrum disorders with a variable that modelled large, moderate or small absolute differences in parental age. While they found no significant differences after adjusting for seven covariates, we cannot directly compare their results to ours as their variable did not separate parental age differences in both directions (i.e. younger mothers and older fathers and vice versa). In another study, Buizer-Voskamp *et al.* [50] adjusted for the age difference between parents, but did not report those results.

Our study shows that autistic and schizophrenic disorder risks tend to be higher, in more dissimilarly aged parents, relative to parents with the most similar ages. However, autism risk plateaus towards the extreme parental age differences, i.e. between parental age difference groups 5–6 (i.e. fathers 11–27 years older than mothers) and 1–2 (i.e. mothers 4–14 years older than fathers) (Fig. 1C). This suggests that there may be protective effects for autism conferred from younger maternal (i.e. 15–28 years) and paternal (i.e. 16–30 years) age groups 1–3 (Fig. 1A and B) that neutralize the negative effects of the older parent. This highlights the complexity of psychiatric disorders when they are affected by both parent’s age and their difference, amongst many other factors within a single generation. Nonetheless, our consistently significant results for parental age difference suggests that this factor needs to be accounted for in future studies of mental disorder risks when data sets are large enough to allow this.

### Higher schizophrenic risks in offspring trend to higher autistic risks as mothers age

The overall picture offered by Fig. 1A and B/D and E is that schizophrenia risks are highest, when parents (particularly mothers) are youngest, but these risks appear to fade away towards the middle of the reproductive period and start trending towards ever-larger autistic disorder risks as mothers and their partners age. This pattern can be interpreted using a combination of POC theory, PC theory and LH theory as summarized in Introduction section. These conceptual frameworks coalesce in the recently developed IB theory [29, 60, 61], which hypothesizes that autism and schizophrenia are the extremes of a single perturbation gradient between paternal and maternal reproductive interests. As described in the introduction, POC and LH theory predict a gradient of high to low resource conflict between mother and offspring when her declining reproductive value weakens selection on maternal genes to withhold resources from focal offspring as later offspring become less likely. In support of this, older mothers tend to give birth to larger babies [62], are more attentive on average, have less conflict with their offspring [63], and see them grow up with fewer hospital visits and better than average general health and development [64]. Our data also revealed that maternal age is significantly positively correlated with essentially all relevant birth-size traits (Supplementary Table S15). Such differences in offspring quality (i.e. health, survival) as well as a statistically decreasing rate of partner change with women’s age [65–67] could all contribute to resource allocation during and after pregnancy being less constrained in older mothers.

The shifting risk patterns with maternal age suggest there may be epigenetic maternal mechanisms that control patri/matrigenic parent-of-origin effects on foetal growth or offspring brain development. This would be consistent with a recent study showing that methylation levels, in particular for functions related to neurological regulation in newborns were significantly associated with maternal (and to a lesser extent paternal) age [68]. Also another study showing that older maternal age is significantly associated with reduced methylation of specific CpG sites [69] may point in this direction. These findings suggest that there are no substantial constraints for the evolution of proximate mechanisms that allow maternal adjustment of patri/matrigenic effects in offspring when there is selection for such regulation because it enhances maternal life-time fitness. Substantial further work will be needed to corroborate the functionality of these putative mechanisms. Age-specific maternal regulation of offspring provisioning may well have been adaptive in ancestral human populations where mothers went though many pregnancies and gradual relaxation of resistance to patrigenic pressure for higher provisioning demands may have been appropriate as they progressed from first to final pregnancy. Too few generations have passed to expect naturally selected ancestral life-history traits to have disappeared in modern humans, so maternal age appears to be a logical ultimate predictor of offspring risk of schizophrenia and autism, independently of birth weight [31].

Explanations of this kind are fundamentally different from proximate factors such as mutation accumulation in gametes or the presence of epigenetic methylation mechanisms that can respond to selection. Mutational load is much more likely for sperm than for eggs given the vast difference in number of cell divisions in testes and ovaries after conception. For such proximate factors it is valid to ask why natural selection has not removed part of these liabilities in our ancestors and the simple answer may be that older parents were never the norm until very recently. This may also explain why increased aneuploidy of eggs released by older mothers has not been a significant risk factor earlier in human history, whereas it may be now.

### A graphical model to facilitate the evolutionary interpretation of mental disorder risks

**Figure 2.**
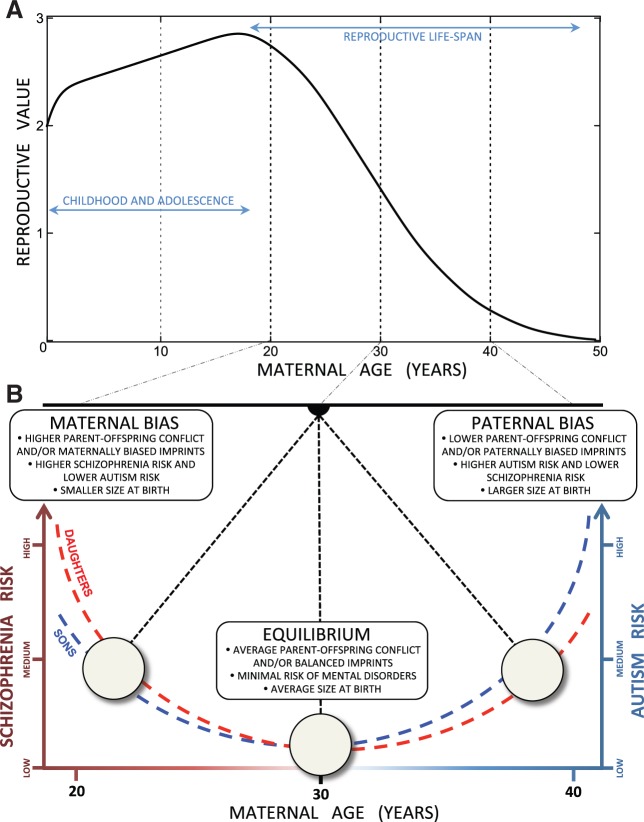
Diagrammatic model of how diametrically opposite risks of mental disorders in offspring can be conceptualized to pivot between schizophrenia and autism as maternal age increases. (**A**) A 1911 reproductive value curve for Australian women drawn after R.A. Fisher’s version in his chapter on the fundamental theorem of natural selection [70], specifying that first births occurred at age ∼20 and last births normally at ages just beyond 40, and assuming that maternal median age at birth was ∼30 years. (**B**) The shift from maximal schizophrenia risk and minimal autism risk in offspring born to young mothers on the left, via minimal risk (zero when scaled relative to risk in offspring of median-aged mothers in the population) for any psychiatric disorder in offspring born to mothers of median reproductive value, to maximal autism risk and minimal schizophrenia risk in offspring born to mothers approaching menopause on the right, based on the overall patterns plotted in Fig. 1 and previously documented diametrically opposed risks of autism and schizophrenia dependent on size at birth [31]. Differences in risk related to offspring being daughters or sons ([29]; see Supplementary Tables S5–S14 for documentation) are likely to be minor compared to the effects of maternal age-dependent patri/matrigenically induced provisioning biases and/or maternal genes whose expression is assumed to covary with age to express initially high but gradually diminishing resistance to patrigenic coercion for higher offspring provisioning in the womb and after birth

We have combined the theoretical expectations and overall trends in our findings in a diagram (Fig. 2) to express how mental disorder risks can be mapped on maternal age at birth. We use the established evolutionary concept of Fisherian reproductive value [70], which is maximal shortly after menarche and declines to zero just before menopause. We used Fisher’s own diagram–constructed ca. a century ago before the onset of modern birth control–that is likely to be fairly representative for ancestral female fertility, with first child birth at ∼20 years old and last child birth at just beyond 40 years old. In that interval, reproductive value declines monotonically such that median reproductive value was reached at ∼30 years old, on average after the first three pregnancies. This age represented the majority of mothers from a typical breeding population–experienced in child rearing and not yet affected by senescence. If we assume that patrigenic and matrigenic effects were optimally balanced in these median-age pregnancies, LH theory would imply that no maternal regulation is needed at this stage, whereas younger mothers were selected to resist patrigenic coercion by the foetus or placenta (decreasingly so from first to 3–4th pregnancy) and older mothers to gradually comply with patrigenic efforts for maximal investment in final pregnancies.

Against this background, we would then expect offspring born to median-aged mothers to have average sizes at birth and minimal risks of any mental disorders later in life. Extensions towards both sides can then be represented by a pendulum with young high-reproductive-value mothers on the left and old low-reproductive-value mothers towards the right. That pendulum then swings around a relative mental disorder risk enhancement of zero in the middle, with increasing risks of schizophrenia to the left and increasing autism risks to the right. Our previous study [31] showed that birth sizes below and above average independently predict enhanced and reduced risks of either schizophrenia or autism as indicated in the labels inserted in Fig. 2. Both that study and our present one identified these risks to also be modified by the sex of the offspring, as indicated by the red and blue curves in Fig. 2, consistent with the general mammalian pattern of somewhat higher maternal investment in individual sons than daughters [39, 71], which independently contributes to the birth weight differences. These inferences suggest that it would be useful to extend the two previously identified predictive evolutionary ‘axes of cognition’ [29, 31], the sex of the foetus and the extent of perturbation of the balance between patri- and matrigenic effects, with a third maternal age axis to arrive at a more complete conceptual framework for understanding the ultimate causation of the full spectrum of mental disorders.

We realize that at this point in time Fig. 2 diagram remains informed speculation even though it is based on fairly strong inferences using established evolutionary theory and consistently supported by the public health data that we analyzed. It would therefore be highly desirable that other large-scale public health studies replicate our statistical analyses of the Danish public health data. In addition, no evolutionary predictions or supportive correlations will be fully credible until proximate mechanisms mediating the expression of parental and POCs have been identified and functionally understood. As we made explicit in our previous study [31], the trends that we predicted and statistically documented are all additional to any directly causal heritable variants that enhance or reduce individual risks of being diagnosed with autism- or schizophrenia-spectrum disorders. The massive ongoing research effort to identify directly causal genetic risk variants is thus unlikely to shed proximate light on the relationships that we documented here. The search for genetic mechanisms that may further explain the trends that we documented are likely to be found in conditionally expressed maternal genes that differentially affect placental or foetal functioning, which represents an area of study that appears to have barely begun [45, 68, 69].

### FUNDING

The Centre for Social Evolution and its Evolutionary Medicine program were funded by a grant from the Danish National Research Foundation to J.J. Boomsma. (DNRF57). S. G. Byars was also funded by a Marie Curie International Incoming Fellowship FP7-PEOPLE-2010-IIF-276565.

**Conflict of interest**: None declared

## ETHICS

Approval for our study was obtained from the Danish Data Protection Agency, the Danish National Board of Health, the Danish Psychiatric Central Research Registry, and Statistics Denmark.

## DATA ACCESSIBILITY

Data for this study were made available by public authorities in accordance with The Danish Act on Processing of Personal Data (Act No. 429 of 31 May 2000). Data have been deposited under terms of a contract at Statistics Denmark (www.dst.dk; last accessed August 2016) and data cannot leave the servers at Statistics Denmark. Access to the data used in the present study can be granted to other researchers through an affiliation with the Centre for Social Evolution, University of Copenhagen, if approved by Statistics Denmark. For further information please contact Professor Jacobus J Boomsma, Centre for Social Evolution (JJBoomsma@bio.ku.dk) and the Head of Division for Research Services, Ivan Thaulow (ITH@DST.dk), Statistics Denmark.

## SUPPLEMENTARY DATA

Supplementary data is available at *EMPH* online.

## Supplementary Material

Supplementary Data
