# Supplementary material for: Opposite differential risks for autism and schizophrenia based on maternal age, paternal age, and parental age differences
Source: Evol Med Public Health. 2016 Aug 16;2016(1):286–98. doi: 10.1093/emph/eow023 (PMC5026125; doi:10.1093/emph/eow023)
Supplement: Supplementary Data [file eow023_Supp.zip › eow023-suppl_data/Supplementary_Tables_S1-S15.pdf]

Supporting Information: Tables

Table S1. Summary of recent studies testing the effect of maternal and paternal age on risk of autism and schizophrenia

	sample location[a]	number of psychiatric cases	Range of years offspring born	number of years offspring followed	offspring age range last observed	ICD coding system used	number of disorders considered	focal disorder	other parent[b]	parity[b]	offspring sex[b]	family psychiatric history[b]	parental education[b]	birth weight or length[b]	gestation length[b]	parental income[b]	race/ethnicity[b]	offspring birth year[b]	offspring birth season[b]	parental occupation[b]	urbanicity[b]	total number of covariates used	risk in youngest parental age group[c]	risk in oldest parental age group[d]
**AUTISM**																								
McGrath 2014 [1]	DK		93-06	16	5-19	10	19	>1	y		y								y			3	M	F
Frans 2013 [2]	SE	5933		22		9/10	1	A	y		y	y	y					y		y		7	M*	F,M*
Lampi 2013 [3]	FI	4713	87-05	20	2-21	9/10	1	ASD		y		y		y	y					y		6	F*, M*	F*, M*
van Balkom 2012 [4]	AR	95	90-03	16	3-16	DSM-IV	1	A	y		y			y								5		F*
Parner 2012 [5]	DK	9556	80-03	29	6-29	8/10	1	ASD		y	y	y		y	y							5		F,M
Hultman 2011 [6]	SE	883	83-92	?	10-19	9/10	1	A	y	y				y	y			y				9		F
Lundstrom 2010 [7]	SE, UK	13	92-98		8-12	10	1	ASD	y		y		y									4	F*	F*
Shelton 2010 [8]	U.S.	12159	90-99	?	6		1	A	y	y			y				y	y				6		M,F
Sasanfar 2010 [9]	IR	179	94-00	?	5-11	DSM-IV	1	A		y	y		y								y	6		F*
**SCHIZOPHRENIA**																								
McGrath 2014 [1]	DK		93-06	16	5-19	10	19	>1	y		y								y			3	M	F
Miller 2011 [10]	many							SCZ,PSY														0	F*	F*
Jaffe 2014 [11]	U.S.			15			1	SCZ	y	y												3		M,F*
Wu 2012 [12]	CN	351	58-03	3	25(7.6)		2	SCZ	y		y											2	M	F
Buizer-Voskamp 2011 [13]	NL	14231	47+	9	36(9)	DSM-IV	4	SCZ,MD,BP								y	y					4		F*
Byrne 2003 [14]	DK	7704		17	15+	8/10	1	SCZ	y	y		y				y						7	F*,M*	F, M*
Rasmussen 2006 [15]	SE	736	73-80	?	22-29	9/10	1	SCZ	y	y	y	y	y	y	y	y			y		y	14		F
Zammit 2003 [16]	SE	362		26		8	1	SCZ	y													5		F
Brown 2002 [17]	U.S.	68	59-67	?	14-38	9	2	SCZ, SSD	y	y			y				y					4		F*
Tsuchiya 2005 [18]	JP	99						SCZ	y	y	y	y										4		F
Wohl 2006 [19]	many							SCZ														0	F	F
El-Saadi 2004 [20]	many	310				DSM-III		PSY														0	M*	F*

Supporting Information: Tables

Table S1 footnotes:
[a] Country code; Aruba (AR), China (CN), Denmark (DK), Finland (FI), Iran (IR), Japan (JP), Netherlands (NL), Sweden (SE), United Kingdom (UK), United States (US), many (many countries considered)
[b] Covariates adjusted for in the analysis (y=yes)
[c] Significant risk detected in the youngest parental age group (M, F, significant for maternal or paternal age, respectively)
[d] Significant risk detected in the oldest parental age group (M, F, significant for maternal or paternal age, respectively)
[*] Indicates that this effect was not consistent, i.e. it disappeared depending on what covariates were adjusted for

Table S1 References

1. McGrath, J.J., et al., *A comprehensive assessment of parental age and psychiatric disorders.* JAMA Psychiatry, 2014. **71**(3): p. 301-9.
2. Frans, E.M., et al., *Autism Risk Across Generations A Population-Based Study of Advancing Grandpaternal and Paternal Age.* Jama Psychiatry, 2013. **70**(5): p. 516-521.
3. Lampi, K.M., et al., *Parental Age and Risk of Autism Spectrum Disorders in a Finnish National Birth Cohort.* Journal of Autism and Developmental Disorders, 2013. **43**(11): p. 2526-2535.
4. van Balkom, I.D.C., et al., *Paternal Age and Risk of Autism in an Ethnically Diverse, Non-Industrialized Setting: Aruba.* Plos One, 2012. **7**(9).
5. Parner, E.T., et al., *Parental Age and Autism Spectrum Disorders.* Annals of Epidemiology, 2012. **22**(3): p. 143-150.
6. Hultman, C.M., et al., *Advancing paternal age and risk of autism: new evidence from a population-based study and a meta-analysis of epidemiological studies.* Molecular Psychiatry, 2011. **16**(12): p. 1203-1212.
7. Lundstrom, S., et al., *Trajectories leading to autism spectrum disorders are affected by paternal age: findings from two nationally representative twin studies.* Journal of Child Psychology and Psychiatry, 2010. **51**(7): p. 850-856.
8. Shelton, J.F., D.J. Tancredi, and I. Hertz-Picciotto, *Independent and Dependent Contributions of Advanced Maternal and Paternal Ages to Autism Risk.* Autism Research, 2010. **3**(1): p. 30-39.
9. Sasanfar, R., et al., *Paternal age increases the risk for autism in an Iranian population sample.* Mol Autism, 2010. **1**(1): p. 2.
10. Miller, B., et al., *Meta-analysis of paternal age and schizophrenia risk in male versus female offspring.* Schizophr Bull, 2011. **37**(5): p. 1039-47.
11. Jaffe, A.E., et al., *Paternal age, de novo mutations and schizophrenia.* Molecular Psychiatry, 2014. **19**(3): p. 274-275.
12. Wu, Y., et al., *Advanced paternal age increases the risk of schizophrenia and obsessive-compulsive disorder in a Chinese Han population.* Psychiatry Res, 2012. **198**(3): p. 353-9.
13. Buizer-Voskamp, J.E., et al., *Paternal age and psychiatric disorders: Findings from a Dutch population registry.* Schizophrenia Research, 2011. **129**(2-3): p. 128-132.
14. Byrne, M., et al., *Parental age and risk of schizophrenia: a case-control study.* Arch Gen Psychiatry, 2003. **60**(7): p. 673-8.
15. Rasmussen, F., *Paternal age, size at birth, and size in young adulthood - risk factors for schizophrenia.* European Journal of Endocrinology, 2006. **155**: p. S65-S69.
16. Zammit, S., et al., *Paternal age and risk for schizophrenia.* Br J Psychiatry, 2003. **183**: p. 405-8.

17. Brown, A.S., et al., *Paternal age and risk of schizophrenia in adult offspring.* Am J Psychiatry, 2002. **159**(9): p. 1528-33.
18. Tsuchiya, K.J., et al., *Advanced paternal age associated with an elevated risk for schizophrenia in offspring in a Japanese population.* Schizophr Res, 2005. **76**(2-3): p. 337-42.
19. Wohl, M. and P. Gorwood, *Paternal ages below or above 35 years old are associated with a different risk of schizophrenia in the offspring.* European Psychiatry, 2007. **22**(1): p. 22-26.
20. El-Saadi, O., et al., *Paternal and maternal age as risk factors for psychosis: findings from Denmark, Sweden and Australia.* Schizophr Res, 2004. **67**(2-3): p. 227-36.

Supporting Information: Tables

Table S2. Characteristics of the study samples for autism- and schizophrenia-spectrum disorder groups. The autism- and schizophrenia-spectrum columns give the characteristics specifically for cases, whereas the total sample columns to the right of each combine case (n=10,703 and 20,586 for autism- and schizophrenia-spectrum diagnosed individuals, respectively) and control (n=1,646,092) individuals (e.g. 10,703+1,646,092=1,656,795). Numbers (n), means (μ), percentages (%, binary traits multiplied by 100, so they are percentages) and Standard Deviations (SD, in brackets) are provided. Absolute risks for autism- and schizophrenia-spectrum disorders within the study period were 0.65% and 1.23%, respectively. Note, study characteristics for the other eight disorder groups (defined in Materials and Methods) are not shown here.

	**autism-spectrum**	**total sample**	**schizophrenia-spectrum**	**total sample**
offspring with disorder (**n**)	10,703	1,656,795	20,586	1,666,678
(%)	0.65 (0.08)	-	1.23 (0.11)	-
mothers with same disorder (%)	0.13 (0.03)	0.008 (0.009)	9.14 (0.28)	3.23 (0.17)
fathers with same disorder (%)	0.21 (0.04)	0.014 (0.01)	5.95 (0.23)	2.24 (0.14)
paternal age group (%)				
group 1 (16 to 20 yrs)	0.53 (0.07)	0.55 (0.07)	1.19 (0.10)	0.56 (0.07)
group 2 (21 to 25 yrs)	9.20 (0.28)	9.28 (0.29)	16.59 (0.37)	9.37 (0.29)
group 3 (26 to 30 yrs)	29.22 (0.45)	30.49 (0.46)	34.07 (0.47)	30.54 (0.46)
central (31 to 34 yrs)	27.00 (0.44)	28.43 (0.45)	23.84 (0.42)	28.39 (0.45)
group 4 (35 to 39 yrs)	21.32 (0.41)	20.88 (0.40)	16.02 (0.36)	20.81 (0.40)
group 5 (40 to 44 yrs)	8.63 (0.28)	7.46 (0.26)	5.83 (0.23)	7.43 (0.26)
group 6 (45 to 60 yrs)	4.08 (0.19)	2.88 (0.16)	2.42 (0.15)	2.87 (0.16)
maternal age group (%)				
group 1 (15 to 21 yrs)	4.25 (0.20)	4.25 (0.20)	9.37 (0.29)	4.31 (0.20)
group 2 (22 to 24 yrs)	11.54 (0.32)	10.95 (0.31)	18.60 (0.38)	11.04 (0.31)
group 3 (25 to 28 yrs)	26.95 (0.44)	27.53 (0.44)	30.35 (0.46)	27.57 (0.44)
central (29 to 31 yrs)	22.79 (0.42)	23.63 (0.42)	18.78 (0.39)	23.58 (0.42)
group 4 (32 to 34 yrs)	17.41 (0.37)	17.67 (0.38)	12.50 (0.33)	17.61 (0.38)
group 5 (35 to 38 yrs)	12.33 (0.32)	11.96 (0.32)	7.90 (0.27)	11.91 (0.32)
group 6 (39 to 46 yrs)	4.69 (0.21)	3.97 (0.19)	2.46 (0.15)	3.94 (0.19)
parental age difference group (%)				
group 1 (-14 to -8 yrs)	0.48 (0.07)	0.42 (0.06)	0.57 (0.07)	0.42 (0.06)
group 2 (-7 to -4 yrs)	2.80 (0.16)	2.26 (0.14)	2.29 (0.15)	2.26 (0.14)
group 3 (-3 to 1 yrs)	25.20 (0.43)	26.18 (0.44)	22.80 (0.42)	26.14 (0.43)
central (2 to 6 yrs)	47.97 (0.50)	51.12 (0.50)	51.82 (0.50)	51.15 (0.50)
group 4 (7 to 10 yrs)	14.51 (0.35)	13.53 (0.34)	14.37 (0.35)	13.54 (0.34)
group 5 (11 to 15 yrs)	6.59 (0.24)	4.76 (0.21)	5.73 (0.23)	4.77 (0.21)
group 6 (16 to 27 yrs)	2.41 (0.15)	1.68 (0.12)	2.39 (0.15)	1.69 (0.12)
pre-existing hypertension (%)	0.23 (0.04)	0.32 (0.05)	0.17 (0.04)	0.32 (0.05)
pre-existing diabetes (%)	0.47 (0.06)	0.38 (0.06)	0.34 (0.05)	0.38 (0.06)
previous induced abortion (%)	20.87 (0.40)	17.98 (0.38)	21.49 (0.41)	18.00 (0.38)
previous spontaneous abortion (%)	15.61 (0.36)	14.28 (0.35)	16.16 (0.36)	14.29 (0.35)
education, combined total yrs (μ)	25.61 (4.64)	25.74 (4.72)	24.24 (4.99)	25.72 (4.73)
income, combined average Dkr. (μ)	351924 (136455)	366390 (172683)	385072 (168608)	366714 (172850)
gestation length, weeks (μ)	39.64 (1.78)	39.76 (1.64)	39.66 (1.67)	39.76 (1.63)
maternal bleeding (%)	8.56 (0.28)	7.35 (0.26)	8.64 (0.28)	7.36 (0.26)
fetal oxygen deprivation (%)	0.04 (0.02)	0.08 (0.03)	0.24 (0.05)	0.09 (0.03)
pregnancy oedema (%)	0.63 (0.07)	0.80 (0.08)	1.44 (0.11)	0.81 (0.09)
gestational diabetes (%)	0.22 (0.04)	0.35 (0.06)	<0.01 (<0.01)	0.35 (0.05)
gestational hypertension (%)	4.90 (0.21)	3.77 (0.19)	4.63 (0.21)	3.78 (0.19)
birth weight, g (μ)	3501.13 (487.4)	3488.13 (457.8)	3392.57 (454.3)	3486.87 (457.7)
APGAR 5 score, 1-10 (μ)	9.81 (0.80)	9.87 (0.62)	9.88 (0.61)	9.87 (0.61)
sex, 0=male, 1=female (%)	19.57 (0.39)	48.45 (0.50)	64.33 (0.47)	48.84 (0.50)
birth year (μ)	1993.95 (5.98)	1994.12 (8.59)	1984.80 (4.48)	1994.01 (8.63)
birth season, months 1-12 (μ)	6.48 (3.37)	6.46 (3.36)	6.39 (3.38)	6.46 (3.36)
nationality, 0=Danish, 1=other (%)	5.29 (0.22)	6.91 (0.25)	2.58 (0.15)	6.87 (0.25)

Supporting Information: Tables

demographic parity (%)				
first born	49.87 (0.50)	44.05 (0.49)	45.27 (0.49)	44.03 (0.49)
second born	34.14 (0.47)	37.53 (0.48)	36.44 (0.48)	37.54 (0.48)
third born	11.99 (0.32)	13.76 (0.34)	13.51 (0.34)	13.76 (0.34)
fourth (or higher born)	3.98 (0.19)	4.64 (0.21)	4.75 (0.21)	4.65 (0.21)
region in Denmark most lived (%)				
Hovedstaden	36.11 (0.48)	28.42 (0.45)	27.46 (0.44)	28.36 (0.45)
Sjælland	13.02 (0.33)	14.73 (0.35)	13.17 (0.33)	14.72 (0.35)
Syddanmark	18.24 (0.38)	22.25 (0.41)	24.95 (0.43)	22.31 (0.41)
Midtjylland	23.73 (0.42)	23.73 (0.42)	26.53 (0.44)	23.76 (0.42)
Nordjylland	8.88 (0.28)	10.85 (0.31)	7.86 (0.26)	10.82 (0.31)

Supporting Information: Tables

Table S3. ICD-8 and ICD-10 classification codes used for autism, schizophrenia and related disorders.

	**AUTISTIC**	
**autism** - infantile	**ICD-8:**	299.00
	**ICD10:**	F84.0
**autism** - all types (infantile and atypical autism)	**ICD-8:**	299.00, 299.01
	**ICD10:**	F84.0, F84.1, F84.10, F84.11, F84.12
**autism-spectrum disorders** – infantile and atypical autism, Asperger's syndrome, pervasive developmental disorder, not otherwise specified (PDD-NOS)	**ICD-8:**	299.00, 299.01, 299.02, 299.03
	**ICD10:**	F84.0, F84.1, F84.10, F84.11, F84.12, F84.5, F84.8, F84.9
**disorders of psychological development**	**ICD-8:**	299.00, 299.01, 299.02, 299.03, 306.09, 306.10, 306.11, 306.12, 306.18, 306.19, 306.39
	**ICD10:**	F80, F80.0, F80.1, F80.2, F80.3, F80.8, F80.9, F81.0, F81.1, F81.2, F81.3, F81.8, F81.9, F82, F82.9, F83, F83.9, F84, F84.0, F84.1, F84.10, F84.11, F84.12, F84.2, F84.3, F84.4, F84.5, F84.8, F84.9, F88, F88.9, F89, F89.9
**behavioral and emotional disorders with onset in childhood and adolescence**	**ICD-8:**	308.00, 308.01, 308.02, 308.03, 308.04, 308.05, 308.06, 308.07, 308.09, 306.29, 306.49, 306.50, 306.58, 306.59
	**ICD10:**	F90, F90.0, F90.1, F90.8, F90.9, F91, F91.0, F91.1, F91.2, F91.3, F91.8, F91.9, F92, F92.0, F92.8, F92.9, F93, F93.0, F93.1, F93.2, F93.3, F93.8, F93.80, F93.9, F94, F94.0, F94.1, F94.2, F94.8, F94.9, F95.0, F95.1, F95.2, F95.8, F95.9, F98, F98.0, F98.00, F98.01, F98.02, F98.1, F98.10, F98.11, F98.12, F98.2, F98.3, F98.4, F98.40, F98.41, F98.42, F98.5, F98.8, F98.8C, F98.9
	**SCHIZOPHRENIC**	
**schizophrenia**	**ICD-8:**	295.09, 295.19, 295.29, 295.39, 295.59, 295.69, 295.89, 295.99
	**ICD10:**	F20, F20.0, F20.00, F20.01, F20.02, F20.03, F20.04, F20.05, F20.06, F20.07, F20.08, F20.09, F20.1, F20.10, F20.11, F20.12, F20.13, F20.14, F20.15, F20.18, F20.19, F20.2, F20.20, F20.21, F20.22, F20.23, F20.24, F20.25, F20.28, F20.29, F20.3, F20.30, F20.31, F20.32, F20.33, F20.34, F20.35, F20.38, F20.39, F20.4, F20.40, F20.44, F20.5, F20.50, F20.51, F20.52, F20.53, F20.54, F20.55, F20.58, F20.59, F20.6, F20.60, F20.61, F20.62, F20.63, F20.64, F20.65, F20.69, F20.8, F20.80, F20.81, F20.84, F20.88, F20.9, F20.90, F20.91, F20.92, F20.93, F20.94, F20.95, F20.98, F20.99
**bipolar disorder**	**ICD-8:**	296.19, 296.39
	**ICD10:**	F30, F30.0, F30.1, F30.2, F30.20, F30.21, F30.8, F30.9, F31, F31.0, F31.1, F31.2, F31.20, F31.21, F31.3, F31.30, F31.31, F31.4, F31.5, F31.50, F31.51, F31.6, F31.7, F31.8, F31.9
**major depression**	**ICD-8:**	296.09, 296.29, 296.99, 298.09, 300.49
	**ICD10:**	F32, F32.0, F32.00, F32.01, F32.1, F32.10, F32.11, F32.2, F32.3, F32.30, F32.31, F32.8, F32.9, F32.9A, F33, F33.0, F33.00, F33.01, F33.1, F33.10, F33.11, F33.2, F33.21, F33.3, F33.30, F33.31, F33.4, F33.8, F33.9

Supporting Information: Tables

**schizophrenic-spectrum disorders** - schizophrenia, bipolar, major depression	**ICD-8:**	295.09, 295.19, 295.29, 295.39, 295.59, 295.69, 295.89, 295.99, 296.19, 296.39, 296.09, 296.29, 296.99, 298.09, 300.49
	**ICD10:**	F20, F20.0, F20.00, F20.01, F20.02, F20.03, F20.04, F20.05, F20.06, F20.07, F20.08, F20.09, F20.1, F20.10, F20.11, F20.12, F20.13, F20.14, F20.15, F20.18, F20.19, F20.2, F20.20, F20.21, F20.22, F20.23, F20.24, F20.25, F20.28, F20.29, F20.3, F20.30, F20.31, F20.32, F20.33, F20.34, F20.35, F20.38, F20.39, F20.4, F20.40, F20.44, F20.5, F20.50, F20.51, F20.52, F20.53, F20.54, F20.55, F20.58, F20.59, F20.6, F20.60, F20.61, F20.62, F20.63, F20.64, F20.65, F20.69, F20.8, F20.80, F20.81, F20.84, F20.88, F20.9, F20.90, F20.91, F20.92, F20.93, F20.94, F20.95, F20.98, F20.99, F30, F30.0, F30.1, F30.2, F30.20, F30.21, F30.8, F30.9, F31, F31.0, F31.1, F31.2, F31.20, F31.21, F31.3, F31.30, F31.31, F31.4, F31.5, F31.50, F31.51, F31.6, F31.7, F31.8, F31.9, F32, F32.0, F32.00, F32.01, F32.1, F32.10, F32.11, F32.2, F32.3, F32.30, F32.31, F32.8, F32.9, F32.9A, F33, F33.0, F33.00, F33.01, F33.1, F33.10, F33.11, F33.2, F33.21, F33.3, F33.30, F33.31, F33.4, F33.8, F33.9
**schizophrenia-schizotypal-delusional disorders**	**ICD-8:**	295.09, 295.19, 295.29, 295.39, 295.59, 295.69, 295.89, 295.99, 297.09, 297.19, 297.99, 298.39, 296.89, 301.83
	**ICD10:**	F20, F20.0, F20.00, F20.01, F20.02, F20.03, F20.04, F20.05, F20.06, F20.07, F20.08, F20.09, F20.1, F20.10, F20.11, F20.12, F20.13, F20.14, F20.15, F20.18, F20.19, F20.2, F20.20, F20.21, F20.22, F20.23, F20.24, F20.25, F20.28, F20.29, F20.3, F20.30, F20.31, F20.32, F20.33, F20.34, F20.35, F20.38, F20.39, F20.4, F20.40, F20.44, F20.5, F20.50, F20.51, F20.52, F20.53, F20.54, F20.55, F20.58, F20.59, F20.6, F20.60, F20.61, F20.62, F20.63, F20.64, F20.65, F20.69, F20.8, F20.80, F20.81, F20.84, F20.88, F20.9, F20.90, F20.91, F20.92, F20.93, F20.94, F20.95, F20.98, F20.99, F21, F21.0, F21.00, F21.9, F22, F22.0, F22.00, F22.2, F22.8, F22.9, F25, F25.0, F25.00, F25.01, F25.1, F25.10, F25.11, F25.2, F25.20, F25.21, F25.8, F25.80, F25.81, F25.9, F25.90, F25.91, F28, F28.0, F28.9, F29, F29.9

Supporting Information: Tables

Table S4. ICD-8 and ICD-10 classification codes used for covariates.

	**PRE-EXISTING CONDITIONS**	
**pre-existing maternal hypertension** – essential (primary) hypertension, hypertensive heart disease, hypertensive renal disease, hypertensive heart and renal disease, secondary hypertension	**ICD-8:**	400.09, 400.19, 400.29, 400.39, 400.99, 401.99, 402.99, 403.99, 404.99
	**ICD10:**	O10, O10.0, O10.0A-0C, O10.1, O10.1A-1C, O10.2, O10.2A-2C, O10.3, O10.3A, O10.4, O10.4A-4C, O10.9, O10.9A-9C, O11, O11.9, O11.9A-9C, I10, I10.9, I11, I11.0, I11.9, I12, I12.0, I12.9, I13, I13.0-2, I13.9, I15, I15.0-2, I15.8-9
**pre-existing maternal diabetes** – type I (insulin dependent) and type II (non-insulin dependent), malnutrition-related, other, unspecified	**ICD-8:**	249.00-09, 250.00-09
	**ICD10:**	E10, E10.0, E10.0A-0F, E10.1-5, E10.5A-5D, E10.6-9, E10.9A, E11, E11.0, E11.0A-0E, E11.1-5, E11.5A-5D, E11.6-9, E11.9A, E12, E12.0, E12.0A-0E, E12.1-5, E12.5A-5C, E12.6-9, E13, E13.0-9, E14, E14.0, E14.0A-0D, E14.1-5, E14.5A-5D, E14.6-9, O24.0, O24.0A-0C, O24.1, O24.1A-1C, O24.2, O24.2A, O24.3, O24.3A-3B
	**PREGNANCY-INDUCED**	
**maternal bleeding** – haemorrhage in early pregnancy, placenta praevia, premature separation of placenta, antepartum haemorrhage	**ICD-8:**	632.09, 632.19, 632.29, 632.39, 632.49, 632.99, 651.00-22, 651.24-43, 651.45-60, 651.63, 651.65-66, 651.68-80, 651.82, 651.85-87, 651.89-99
	**ICD10:**	O20, O20.0, O20.8, O20.8A, O20.8B, O20.9, O44, O44.0-3, O44.9, O45, O45.0, O45.0D, O45.1, O45.1B, O45.2-3, O45.8-9, O46, O46.0, O46.8, O46.8A, O46.9
**fetal oxygen deprivation** – intrauterine hypoxia, birth asphyxia	**ICD-8:**	776.39, 776.49, 776.90-93, 776.98-99
	**ICD10:**	P20, P20.0, P20.0A-0E, P20.1, P20.1A-1B, P20.1D-1E, P20.9, P21, P21.0, P21.0A-0D, P21.1, P21.1A-1D, P21.9
**pregnancy oedema**	**ICD-8:**	637.02
	**ICD10:**	O12, O12.0-2
**gestational diabetes**	**ICD10:**	O24.4D
**gestational hypertension** – pregnancy-induced hypertension, preeclampsia, eclampsia	**ICD-8:**	637.00, 637.03, 637.04, 637.09, 637.19
	**ICD10:**	O13, O13.9, O14, O14.0, O14.1, O14.2, O14.9, O15, O15.0, O15.1, O15.2, O15.9

Supporting Information: Tables

Tables S5-S14. For each table below, the top 18 rows give disorder risk by paternal age, maternal age and parental age difference groups and directly below these are risk ratios for covariates entered into the Cox regressions. Red or blue arrows show significantly increased or decreased risk respectively. Missing coefficients (-) were due to a lack of variation for that variable. P-values were all Bonferroni corrected. LCL, lower 95% confidence limit; UCL, upper 95% confidence limit; SE, standard error of the coefficient.

Table S5. Risk of infantile autism (DF84.0) by paternal age, maternal age and parental age difference at birth.

	exp(coef)	LCL	UCL	risk	SE	Z	Pr(Z)
P age group 1	0.4210	0.2001	0.8857	V	0.3794	-2.2797	0.0226
P age group 2	0.7070	0.5646	0.8854	V	0.1147	-3.0211	0.0025
P age group 3	0.8544	0.7539	0.9684	V	0.0639	-2.4626	0.0138
P age group 4	1.1167	0.9817	1.2703	.	0.0657	1.6792	0.0931
P age group 5	1.2389	1.0035	1.5295	Λ	0.1075	1.9923	0.0463
P age group 6	1.5272	1.1030	2.1147	Λ	0.1661	2.5501	0.0108
M age group 1	0.7685	0.5607	1.0533	.	0.1608	-1.6369	0.1017
M age group 2	0.7639	0.6205	0.9404	V	0.1061	-2.5397	0.0111
M age group 3	0.8453	0.7407	0.9646	V	0.0674	-2.4952	0.0126
M age group 4	1.0029	0.8746	1.1501	.	0.0699	0.0417	0.9667
M age group 5	1.2257	1.0266	1.4634	Λ	0.0904	2.2498	0.0245
M age group 6	1.2156	0.9210	1.6044	.	0.1416	1.3785	0.1681
P-M group 1	1.0854	0.5739	2.0526	.	0.3251	0.2520	0.8010
P-M group 2	1.5706	1.1991	2.0571	Λ	0.1377	3.2786	0.0010
P-M group 3	1.1508	1.0251	1.2920	Λ	0.0590	2.3799	0.0173
P-M group 4	1.0858	0.9446	1.2482	.	0.0711	1.1578	0.2469
P-M group 5	1.4274	1.1467	1.7768	Λ	0.1117	3.1857	0.0014
P-M group 6	1.2922	0.9040	1.8470	.	0.1823	1.4064	0.1596
maternal disorder	-	-	-	.	-	-	-
paternal disorder	-	-	-	.	-	-	-
gestation length	0.9685	0.9481	0.9892	V	0.0108	-2.9604	0.0031
maternal bleeding	1.0557	0.9232	1.2073	.	0.0684	0.7921	0.4283
fetal oxygen deprivation	-	-	-	.	-	-	-
pregnancy oedema	0.9953	0.6245	1.5863	.	0.2378	-0.0197	0.9842
gestational diabetes	1.0754	0.5573	2.0754	.	0.3354	0.2168	0.8283
gestational hypertension	1.1754	0.9784	1.4122	.	0.0936	1.7263	0.0843
APGAR5 score	0.8236	0.7543	0.8992	V	0.0448	-4.3298	<0.0001
birth weight (grams)	0.9998	0.9997	0.9999	V	0.0000	-4.2732	<0.0001
pre-existing hypertension	0.5620	0.2515	1.2556	.	0.4102	-1.4050	0.1600
pre-existing diabetes	1.3322	0.8117	2.1866	.	0.2528	1.1345	0.2566
previous abortion (induc.)	1.0613	0.9670	1.1648	.	0.0475	1.2535	0.2100
previous abortion (spont.)	1.1013	0.9929	1.2216	.	0.0529	1.8255	0.0679
parity (2nd)	0.8969	0.8214	0.9794	V	0.0449	-2.4227	0.0154
parity (3rd)	0.7961	0.7013	0.9038	V	0.0647	-3.5227	0.0004
parity (4+)	0.6723	0.5542	0.8156	V	0.0986	-4.0275	<0.0001

sex	0.2374	0.2158	0.2612	∨	0.0487	-29.5018	<0.0001
education level	0.9720	0.9462	0.9985	∨	0.0137	-2.0719	0.0383
average income	0.8907	0.8755	0.9063	∨	0.0088	-13.1049	<0.0001
nationality	0.9818	0.8571	1.1246	.	0.0693	-0.2652	0.7908
birth year cohort	1.4161	1.3844	1.4485	∧	0.0116	30.1025	<0.0001
birth season	1.0098	0.9987	1.0210	.	0.0056	1.7317	0.0833
region Sjælland	0.8450	0.7584	0.9415	∨	0.0552	-3.0534	0.0023
region Syddanmark	0.5358	0.4801	0.5981	∨	0.0561	-11.1244	<0.0001
region Midtjylland	0.5703	0.5132	0.6338	∨	0.0538	-10.4337	<0.0001
region Nordjylland	0.4779	0.4102	0.5567	∨	0.0779	-9.4756	<0.0001

Table S6. Risk of autism (DF84.0-1) by paternal age, maternal age and parental age difference at birth.

	exp(coef)	LCL	UCL	risk	SE	Z	Pr(Z)
P age group 1	0.4938	0.2792	0.8736	∨	0.2910	-2.4244	0.0153
P age group 2	0.7437	0.6161	0.8978	∨	0.0961	-3.0827	0.0021
P age group 3	0.8915	0.8015	0.9917	∨	0.0543	-2.1125	0.0346
P age group 4	1.0876	0.9732	1.2155	.	0.0567	1.4814	0.1385
P age group 5	1.2453	1.0398	1.4913	∧	0.0920	2.3843	0.0171
P age group 6	1.3511	1.0190	1.7915	∧	0.1439	2.0909	0.0365
M age group 1	0.9123	0.7015	1.1864	.	0.1341	-0.6851	0.4933
M age group 2	0.8817	0.7395	1.0511	.	0.0897	-1.4039	0.1603
M age group 3	0.9251	0.8250	1.0373	.	0.0584	-1.3333	0.1824
M age group 4	1.0667	0.9460	1.2027	.	0.0612	1.0541	0.2918
M age group 5	1.2531	1.0722	1.4645	∧	0.0795	2.8361	0.0046
M age group 6	1.2693	0.9960	1.6177	.	0.1237	1.9273	0.0539
P-M group 1	1.0220	0.5820	1.7947	.	0.2873	0.0758	0.9396
P-M group 2	1.4969	1.1811	1.8971	∧	0.1209	3.3366	0.0008
P-M group 3	1.1202	1.0135	1.2382	∧	0.0511	2.2227	0.0262
P-M group 4	1.0703	0.9491	1.2071	.	0.0614	1.1077	0.2680
P-M group 5	1.4206	1.1761	1.7159	∧	0.0964	3.6436	0.0003
P-M group 6	1.3664	1.0060	1.8558	∧	0.1562	1.9984	0.0457
maternal disorder	21.5262	3.0262	153.121	∧	1.0010	3.0661	0.0022
paternal disorder	-	-	-	.	-	-	-
gestation length	0.9688	0.9515	0.9865	∨	0.0092	-3.4308	0.0006
maternal bleeding	1.0683	0.9530	1.1974	.	0.0582	1.1337	0.2569
fetal oxygen deprivation	0.9244	0.2309	3.7015	.	0.7078	-0.1110	0.9116
pregnancy oedema	1.1353	0.7861	1.6397	.	0.1875	0.6768	0.4985
gestational diabetes	1.2619	0.7140	2.2304	.	0.2906	0.8006	0.4233
gestational hypertension	1.1776	1.0089	1.3746	∧	0.0789	2.0726	0.0382
APGAR5 score	0.8060	0.7490	0.8673	∨	0.0374	-5.7666	<0.0001
birth weight (grams)	0.9999	0.9998	0.9999	∨	0.0000	-3.9983	<0.0001
pre-existing hypertension	0.7200	0.3860	1.3431	.	0.3181	-1.0326	0.3018
pre-existing diabetes	1.3889	0.9184	2.1003	.	0.2110	1.5567	0.1195
previous abortion (induc.)	1.0413	0.9616	1.1275	.	0.0406	0.9951	0.3197
previous abortion (spont.)	1.0968	1.0042	1.1979	∧	0.0450	2.0521	0.0402

parity (2nd)	0.8845	0.8204	0.9536	∨	0.0384	-3.1976	0.0014
parity (3rd)	0.8066	0.7235	0.8992	∨	0.0555	-3.8745	0.0001
parity (4+)	0.7114	0.6030	0.8393	∨	0.0844	-4.0366	<0.0001
sex	0.2737	0.2531	0.2960	∨	0.0399	-32.4692	<0.0001
education level	0.9557	0.9339	0.9780	∨	0.0118	-3.8536	0.0001
average income	0.9010	0.8879	0.9144	∨	0.0075	-13.9105	<0.0001
nationality	0.8544	0.7573	0.9641	∨	0.0616	-2.5537	0.0107
birth year cohort	1.4242	1.3967	1.4523	∧	0.0100	35.5137	<0.0001
birth season	1.0065	0.9970	1.0160	.	0.0048	1.3422	0.1795
region Sjælland	0.7374	0.6704	0.8110	∨	0.0486	-6.2714	<0.0001
region Syddanmark	0.5111	0.4653	0.5615	∨	0.0479	-13.9986	<0.0001
region Midtjylland	0.5974	0.5473	0.6521	∨	0.0447	-11.5229	<0.0001
region Nordjylland	0.4620	0.4058	0.5258	∨	0.0661	-11.6870	<0.0001

Table S7. Risk of autism-spectrum disorders (DF84.0-9) by paternal age, maternal age and parental age difference at birth.

	exp(coef)	LCL	UCL	risk	SE	Z	Pr(Z)
P age group 1	0.7563	0.5648	1.0127	.	0.1490	-1.8751	0.0608
P age group 2	0.8561	0.7682	0.9541	∨	0.0553	-2.8092	0.0050
P age group 3	0.9202	0.8635	0.9805	∨	0.0324	-2.5666	0.0103
P age group 4	1.1044	1.0321	1.1817	∧	0.0345	2.8758	0.0040
P age group 5	1.1857	1.0604	1.3258	∧	0.0570	2.9883	0.0028
P age group 6	1.2464	1.0459	1.4855	∧	0.0895	2.4610	0.0139
M age group 1	0.8268	0.7153	0.9557	∨	0.0739	-2.5723	0.0101
M age group 2	0.9568	0.8679	1.0547	.	0.0497	-0.8888	0.3741
M age group 3	0.9251	0.8670	0.9871	∨	0.0331	-2.3528	0.0186
M age group 4	1.0742	1.0023	1.1512	∧	0.0353	2.0254	0.0428
M age group 5	1.1699	1.0665	1.2834	∧	0.0472	3.3240	0.0009
M age group 6	1.3448	1.1664	1.5505	∧	0.0726	4.0801	<0.0001
P-M group 1	1.1593	0.8611	1.5608	.	0.1517	0.9745	0.3298
P-M group 2	1.3464	1.1705	1.5486	∧	0.0714	4.1653	<0.0001
P-M group 3	1.0938	1.0328	1.1585	∧	0.0293	3.0614	0.0022
P-M group 4	1.1152	1.0406	1.1952	∧	0.0354	3.0851	0.0020
P-M group 5	1.3660	1.2211	1.5280	∧	0.0572	5.4521	<0.0001
P-M group 6	1.2820	1.0656	1.5425	∧	0.0944	2.6329	0.0085
maternal disorder	11.7230	6.9211	19.8564	∧	0.2689	9.1551	<0.0001
paternal disorder	13.1992	8.7456	19.9208	∧	0.2100	12.2860	<0.0001
gestation length	0.9794	0.9687	0.9902	∨	0.0056	-3.7112	0.0002
maternal bleeding	1.0943	1.0217	1.1721	∧	0.0350	2.5730	0.0101
fetal oxygen deprivation	0.6192	0.2575	1.4888	.	0.4476	-1.0709	0.2842
pregnancy oedema	0.8869	0.6980	1.1270	.	0.1222	-0.9819	0.3262
gestational diabetes	1.6938	1.1324	2.5334	∧	0.2054	2.5655	0.0103
gestational hypertension	1.2293	1.1244	1.3440	∧	0.0455	4.5360	<0.0001
APGAR5 score	0.8452	0.8077	0.8844	∨	0.0231	-7.2725	<0.0001
birth weight (grams)	0.9999	0.9999	1.0000	∨	0.0000	-3.4422	0.0006
pre-existing hypertension	0.7567	0.5102	1.1222	.	0.2011	-1.3867	0.1655

pre-existing diabetes	1.1660	0.8837	1.5385	.	0.1414	1.0861	0.2774
previous abortion (induc.)	1.0649	1.0156	1.1166	∧	0.0242	2.5991	0.0093
previous abortion (spont.)	1.0513	0.9966	1.1090	.	0.0273	1.8335	0.0667
parity (2nd)	0.8017	0.7664	0.8386	∨	0.0230	-9.6252	<0.0001
parity (3rd)	0.7111	0.6650	0.7604	∨	0.0342	-9.9610	<0.0001
parity (4+)	0.6037	0.5418	0.6726	∨	0.0551	-9.1550	<0.0001
sex	0.2543	0.2424	0.2669	∨	0.0245	-55.8593	<0.0001
education level	1.0002	0.9861	1.0144	.	0.0072	0.0222	0.9823
average income	0.8941	0.8863	0.9020	∨	0.0045	-25.0090	<0.0001
nationality	0.5452	0.4982	0.5967	∨	0.0461	-13.1712	<0.0001
birth year cohort	1.4053	1.3885	1.4223	∧	0.0061	55.4201	<0.0001
birth season	1.0139	1.0082	1.0196	∧	0.0029	4.8001	<0.0001
region Sjælland	0.6453	0.6066	0.6865	∨	0.0316	-13.8752	<0.0001
region Syddanmark	0.5965	0.5644	0.6305	∨	0.0282	-18.2946	<0.0001
region Midtjylland	0.7451	0.7082	0.7840	∨	0.0260	-11.3312	<0.0001
region Nordjylland	0.5930	0.5519	0.6372	∨	0.0367	-14.2479	<0.0001

Table S8. Risk of disorders of psychological development (DF80-89) by paternal age, maternal age and parental age difference at birth.

	exp(coef)	LCL	UCL	risk	SE	Z	Pr(Z)
P age group 1	0.8374	0.6612	1.0607	.	0.1206	-1.4714	0.1412
P age group 2	0.8931	0.8137	0.9803	∨	0.0475	-2.3782	0.0174
P age group 3	0.9459	0.8951	0.9997	∨	0.0282	-1.9709	0.0487
P age group 4	1.1087	1.0448	1.1765	∧	0.0303	3.4083	0.0007
P age group 5	1.1475	1.0395	1.2668	∧	0.0504	2.7279	0.0064
P age group 6	1.2164	1.0415	1.4207	∧	0.0792	2.4737	0.0134
M age group 1	0.8533	0.7473	0.9743	v	0.0677	-2.3442	0.0191
M age group 2	0.9580	0.8756	1.0481	.	0.0459	-0.9353	0.3497
M age group 3	0.9288	0.8749	0.9860	v	0.0305	-2.4207	0.0155
M age group 4	1.0764	1.0098	1.1474	∧	0.0326	2.2591	0.0239
M age group 5	1.1421	1.0480	1.2446	∧	0.0439	3.0275	0.0025
M age group 6	1.2561	1.0984	1.4366	∧	0.0685	3.3297	0.0009
P-M group 1	1.3518	1.0854	1.6835	∧	0.1120	2.6919	0.0071
P-M group 2	1.4305	1.2862	1.5910	∧	0.0543	6.5996	<0.0001
P-M group 3	1.0630	1.0174	1.1107	∧	0.0224	2.7335	0.0063
P-M group 4	1.1211	1.0637	1.1815	∧	0.0268	4.2646	<0.0001
P-M group 5	1.2805	1.1742	1.3965	∧	0.0442	5.5902	<0.0001
P-M group 6	1.2548	1.0880	1.4472	∧	0.0728	3.1191	0.0018
maternal disorder	6.6463	3.9328	11.2320	∧	0.2677	7.0749	<0.0001
paternal disorder	7.2755	4.8291	10.9612	∧	0.2091	9.4901	<0.0001
gestation length	0.9740	0.9648	0.9833	∨	0.0049	-5.4165	<0.0001
maternal bleeding	1.1194	1.0550	1.1876	∧	0.0302	3.7342	0.0002
fetal oxygen deprivation	0.6573	0.3284	1.3153	.	0.3540	-1.1857	0.2357
pregnancy oedema	0.9889	0.8123	1.2038	.	0.1004	-0.1112	0.9114
gestational diabetes	1.5654	1.0637	2.3038	∧	0.1972	2.2731	0.0230
gestational hypertension	1.1874	1.0975	1.2846	∧	0.0402	4.2750	<0.0001

APGAR5 score	0.8512	0.8180	0.8857	∨	0.0203	-7.9500	<0.0001
birth weight (grams)	0.9999	0.9999	0.9999	∨	0.0000	-4.8705	<0.0001
pre-existing hypertension	0.8085	0.5794	1.1282	.	0.1700	-1.2502	0.2112
pre-existing diabetes	1.3198	1.0530	1.6543	∧	0.1153	2.4076	0.0161
previous abortion (induc.)	1.1087	1.0641	1.1551	∧	0.0209	4.9286	<0.0001
previous abortion (spont.)	1.0582	1.0104	1.1084	∧	0.0236	2.3965	0.0166
parity (2nd)	0.8736	0.8400	0.9085	∨	0.0200	-6.7560	<0.0001
parity (3rd)	0.7880	0.7436	0.8350	∨	0.0296	-8.0591	<0.0001
parity (4+)	0.6704	0.6107	0.7359	∨	0.0476	-8.4088	<0.0001
sex	0.2757	0.2646	0.2872	∨	0.0209	-61.7516	<0.0001
education level	0.9737	0.9617	0.9858	∨	0.0063	-4.2218	<0.0001
average income	0.8971	0.8902	0.9040	∨	0.0039	-27.5912	<0.0001
nationality	0.5157	0.4760	0.5588	∨	0.0409	-16.1981	<0.0001
birth year cohort	1.3393	1.3260	1.3527	∧	0.0051	57.5288	<0.0001
birth season	1.0150	1.0101	1.0200	∧	0.0025	5.9714	<0.0001
region Sjælland	0.6612	0.6251	0.6993	∨	0.0286	-14.4501	<0.0001
region Syddanmark	0.6700	0.6380	0.7035	∨	0.0249	-16.0781	<0.0001
region Midtjylland	0.9826	0.9410	1.0260	.	0.0220	-0.7970	0.4255
region Nordjylland	0.6357	0.5964	0.6776	∨	0.0326	-13.9027	<0.0001

Table S9. Risk of behavioral and emotional disorders (DF90-98) by paternal age, maternal age and parental age difference at birth.

	exp(coef)	LCL	UCL	risk	SE	Z	Pr(Z)
P age group 1	1.1218	0.9854	1.2770	.	0.0661	1.7380	0.0822
P age group 2	1.1245	1.0540	1.1998	∧	0.0331	3.5499	0.0004
P age group 3	1.0061	0.9650	1.0488	.	0.0212	0.2845	0.7760
P age group 4	1.0558	1.0071	1.1069	∧	0.0241	2.2514	0.0244
P age group 5	1.0904	1.0082	1.1792	∧	0.0400	2.1644	0.0304
P age group 6	1.0979	0.9718	1.2405	.	0.0623	1.5000	0.1336
M age group 1	1.3961	1.2993	1.5000	∧	0.0366	9.1043	<0.0001
M age group 2	1.2300	1.1653	1.2983	∧	0.0276	7.5093	<0.0001
M age group 3	1.0865	1.0455	1.1291	∧	0.0196	4.2246	<0.0001
M age group 4	0.9935	0.9504	1.0386	.	0.0226	-0.2882	0.7732
M age group 5	0.9732	0.9170	1.0328	.	0.0303	-0.8971	0.3696
M age group 6	1.0182	0.9276	1.1175	.	0.0475	0.3787	0.7049
P-M group 1	2.0399	1.7217	2.4169	∧	0.0865	8.2390	<0.0001
P-M group 2	1.6453	1.5024	1.8018	∧	0.0464	10.7383	<0.0001
P-M group 3	1.0941	1.0542	1.1356	∧	0.0190	4.7364	<0.0001
P-M group 4	1.2051	1.1527	1.2598	∧	0.0226	8.2360	<0.0001
P-M group 5	1.3530	1.2563	1.4573	∧	0.0379	7.9846	<0.0001
P-M group 6	1.4608	1.2947	1.6483	∧	0.0616	6.1519	<0.0001
maternal disorder	3.6255	3.2716	4.0177	∧	0.0524	24.5766	<0.0001
paternal disorder	2.6453	2.3479	2.9803	∧	0.0608	15.9874	<0.0001
gestation length	0.9652	0.9585	0.9719	∨	0.0035	-9.9819	<0.0001
maternal bleeding	1.2232	1.1729	1.2757	∧	0.0214	9.4064	<0.0001
fetal oxygen deprivation	1.1273	0.7876	1.6136	.	0.1830	0.6549	0.5125

Supporting Information: Tables

pregnancy oedema	1.0613	0.9304	1.2106	.	0.0672	0.8858	0.3757
gestational diabetes	0.9948	0.5883	1.6820	.	0.2680	-0.0196	0.9844
gestational hypertension	1.1320	1.0671	1.2009	∧	0.0301	4.1156	<0.0001
APGAR5 score	0.8914	0.8645	0.9192	∨	0.0156	-7.3468	<0.0001
birth weight (grams)	0.9998	0.9998	0.9999	∨	0.0000	-11.0056	<0.0001
pre-existing hypertension	0.7833	0.5945	1.0320	.	0.1407	-1.7361	0.0825
pre-existing diabetes	1.2649	1.0647	1.5028	∧	0.0879	2.6725	0.0075
previous abortion (induc.)	1.1879	1.1534	1.2235	∧	0.0151	11.4298	<0.0001
previous abortion (spont.)	1.1174	1.0804	1.1558	∧	0.0172	6.4480	<0.0001
parity (2nd)	1.0326	1.0026	1.0635	∧	0.0151	2.1293	0.0332
parity (3rd)	1.0432	0.9992	1.0893	.	0.0220	1.9222	0.0546
parity (4+)	0.9574	0.8956	1.0235	.	0.0340	-1.2775	0.2014
sex	0.4411	0.4294	0.4532	∨	0.0138	-59.2798	<0.0001
education level	0.8718	0.8637	0.8801	∨	0.0048	-28.6306	<0.0001
average income	0.8596	0.8546	0.8646	∨	0.0030	-51.1300	<0.0001
nationality	0.2697	0.2519	0.2888	∨	0.0348	-37.6180	<0.0001
birth year cohort	1.3097	1.3001	1.3194	∧	0.0038	71.7991	<0.0001
birth season	1.0153	1.0117	1.0190	∧	0.0019	8.2122	<0.0001
region Sjælland	0.7573	0.7283	0.7876	∨	0.0200	-13.9136	<0.0001
region Syddanmark	0.7929	0.7662	0.8206	∨	0.0175	-13.2728	<0.0001
region Midtjylland	0.8660	0.8376	0.8954	∨	0.0170	-8.4490	<0.0001
region Nordjylland	0.3964	0.3748	0.4191	∨	0.0285	-32.4635	<0.0001

Table S10. Risk of schizophrenia-spectrum disorders (DF20, DF30, DF31, DF32, DF33) by paternal age, maternal age and parental age difference at birth.

	exp(coef)	LCL	UCL	risk	SE	Z	Pr(Z)
P age group 1	1.0440	0.8877	1.2278	.	0.0827	0.5205	0.6027
P age group 2	1.0800	1.0009	1.1652	∧	0.0388	1.9843	0.0472
P age group 3	1.0101	0.9619	1.0607	.	0.0250	0.4022	0.6875
P age group 4	0.9994	0.9448	1.0571	.	0.0287	-0.0204	0.9837
P age group 5	0.9814	0.8926	1.0791	.	0.0484	-0.3868	0.6989
P age group 6	0.9239	0.7944	1.0745	.	0.0771	-1.0273	0.3043
M age group 1	1.2496	1.1468	1.3615	∧	0.0438	5.0905	<0.0001
M age group 2	1.1225	1.0528	1.1967	∧	0.0327	3.5347	0.0004
M age group 3	1.0056	0.9599	1.0534	.	0.0237	0.2345	0.8146
M age group 4	1.0534	0.9977	1.1122	.	0.0277	1.8761	0.0606
M age group 5	1.0615	0.9865	1.1422	.	0.0374	1.5973	0.1102
M age group 6	0.9942	0.8819	1.1208	.	0.0612	-0.0951	0.9242
P-M group 1	1.2843	1.0490	1.5723	∧	0.1032	2.4232	0.0154
P-M group 2	1.1505	1.0321	1.2825	∧	0.0554	2.5299	0.0114
P-M group 3	0.9998	0.9587	1.0426	.	0.0214	-0.0093	0.9926
P-M group 4	1.0615	1.0098	1.1159	∧	0.0255	2.3431	0.0191
P-M group 5	1.1702	1.0756	1.2731	∧	0.0430	3.6546	0.0003
P-M group 6	1.2938	1.1273	1.4850	∧	0.0703	3.6637	0.0002
maternal disorder	2.3724	2.2533	2.4978	∧	0.0263	32.8804	<0.0001
paternal disorder	1.9892	1.8671	2.1192	∧	0.0323	21.2918	<0.0001

gestation length	0.9910	0.9821	0.9999	∨	0.0046	-1.9767	0.0481
maternal bleeding	1.1733	1.1137	1.2361	∧	0.0266	6.0126	<0.0001
fetal oxygen deprivation	1.0910	0.8188	1.4537	.	0.1464	0.5947	0.5520
pregnancy oedema	1.0006	0.8862	1.1298	.	0.0620	0.0101	0.9919
gestational diabetes	-	-	-	.	-	-	-
gestational hypertension	1.0405	0.9707	1.1152	.	0.0354	1.1206	0.2625
APGAR5 score	0.9599	0.9189	1.0026	.	0.0222	-1.8423	0.0654
birth weight (grams)	0.9999	0.9999	1.0000	∨	0.0000	-3.3202	0.0009
pre-existing hypertension	1.4212	1.0278	1.9653	∧	0.1654	2.1259	0.0335
pre-existing diabetes	1.0186	0.7927	1.3088	.	0.1279	0.1441	0.8854
previous abortion (induc.)	1.1569	1.1165	1.1988	∧	0.0182	8.0303	<0.0001
previous abortion (spont.)	1.0554	1.0140	1.0984	∧	0.0204	2.6445	0.0082
parity (2nd)	1.0686	1.0320	1.1065	∧	0.0178	3.7332	0.0002
parity (3rd)	1.1447	1.0879	1.2044	∧	0.0260	5.2019	<0.0001
parity (4+)	1.1725	1.0836	1.2687	∧	0.0402	3.9555	<0.0001
sex	1.9270	1.8696	1.9861	∧	0.0154	42.5465	<0.0001
education level	1.0050	0.9943	1.0158	.	0.0055	0.9108	0.3624
average income	0.9103	0.9045	0.9160	∨	0.0032	-29.2883	<0.0001
nationality	0.4424	0.4030	0.4858	∨	0.0477	-17.1043	<0.0001
birth year cohort	1.3656	1.3468	1.3847	∧	0.0071	43.9621	<0.0001
birth season	1.0096	1.0053	1.0140	∧	0.0022	4.4013	<0.0001
region Sjælland	0.7723	0.7357	0.8107	∨	0.0248	-10.4378	<0.0001
region Syddanmark	0.9331	0.8962	0.9715	∨	0.0206	-3.3681	0.0008
region Midtjylland	0.9665	0.9289	1.0057	.	0.0203	-1.6778	0.0934
region Nordjylland	0.5652	0.5324	0.5999	∨	0.0304	-18.7529	<0.0001

Table S11. Risk of schizophrenia (DF20) by paternal age, maternal age and parental age difference at birth.

	exp(coef)	LCL	UCL	risk	SE	Z	Pr(Z)
P age group 1	1.0272	0.7433	1.4197	.	0.1651	0.1627	0.8707
P age group 2	0.9620	0.8144	1.1365	.	0.0850	-0.4553	0.6489
P age group 3	0.9399	0.8427	1.0484	.	0.0557	-1.1118	0.2662
P age group 4	0.9563	0.8429	1.0850	.	0.0644	-0.6931	0.4882
P age group 5	1.1250	0.9183	1.3781	.	0.1036	1.1370	0.2555
P age group 6	0.9369	0.6788	1.2932	.	0.1644	-0.3965	0.6917
M age group 1	1.7607	1.4003	2.2139	∧	0.1169	4.8414	<0.0001
M age group 2	1.2998	1.0892	1.5511	∧	0.0902	2.9073	0.0036
M age group 3	1.0733	0.9408	1.2244	.	0.0672	1.0526	0.2925
M age group 4	1.0108	0.8638	1.1828	.	0.0802	0.1339	0.8935
M age group 5	1.0186	0.8264	1.2554	.	0.1067	0.1726	0.8630
M age group 6	0.8857	0.6280	1.2491	.	0.1754	-0.6921	0.4889
P-M group 1	1.7391	1.1544	2.6201	∧	0.2091	2.6466	0.0081
P-M group 2	1.1367	0.8799	1.4685	.	0.1307	0.9810	0.3266
P-M group 3	1.0006	0.9063	1.1046	.	0.0505	0.0114	0.9909
P-M group 4	0.9987	0.8896	1.1212	.	0.0590	-0.0220	0.9825
P-M group 5	1.1439	0.9470	1.3818	.	0.0964	1.3953	0.1629

P-M group 6	1.3983	1.0398	1.8805	∧	0.1512	2.2179	0.0266
maternal disorder	4.4941	3.6890	5.4749	∧	0.1007	14.9202	<0.0001
paternal disorder	3.0165	2.4004	3.7906	∧	0.1165	9.4732	<0.0001
gestation length	0.9976	0.9781	1.0174	.	0.0100	-0.2436	0.8075
maternal bleeding	1.2528	1.1205	1.4006	∧	0.0569	3.9595	<0.0001
fetal oxygen deprivation	1.3157	0.7266	2.3824	.	0.3029	0.9057	0.3651
pregnancy oedema	1.0392	0.8058	1.3402	.	0.1298	0.2966	0.7668
gestational diabetes	-	-	-	.	-	-	-
gestational hypertension	1.0713	0.9216	1.2454	.	0.0768	0.8967	0.3699
APGAR5 score	1.0103	0.9145	1.1162	.	0.0508	0.2019	0.8400
birth weight (grams)	0.9999	0.9998	1.0000	∨	0.0000	-2.5166	0.0118
pre-existing hypertension	0.7857	0.2940	2.0997	.	0.5016	-0.4810	0.6306
pre-existing diabetes	1.2842	0.7713	2.1383	.	0.2601	0.9617	0.3362
previous abortion (induc.)	1.2372	1.1454	1.3364	∧	0.0394	5.4088	<0.0001
previous abortion (spont.)	1.0195	0.9321	1.1151	.	0.0457	0.4222	0.6729
parity (2nd)	1.0303	0.9531	1.1137	.	0.0397	0.7501	0.4532
parity (3rd)	1.1384	1.0174	1.2738	∧	0.0573	2.2616	0.0237
parity (4+)	1.1666	0.9878	1.3777	.	0.0849	1.8159	0.0694
sex	0.7914	0.7416	0.8445	∨	0.0331	-7.0583	<0.0001
education level	1.0259	1.0024	1.0500	∧	0.0118	2.1585	0.0309
average income	0.8481	0.8366	0.8598	∨	0.0070	-23.6252	<0.0001
nationality	0.7350	0.6302	0.8572	∨	0.0785	-3.9223	<0.0001
birth year cohort	1.1818	1.1455	1.2193	∧	0.0159	10.4901	<0.0001
birth season	1.0044	0.9950	1.0140	.	0.0048	0.9168	0.3593
region Sjælland	0.6501	0.5843	0.7234	∨	0.0545	-7.9015	<0.0001
region Syddanmark	0.8321	0.7630	0.9075	∨	0.0442	-4.1544	<0.0001
region Midtjylland	0.7091	0.6478	0.7763	∨	0.0462	-7.4470	<0.0001
region Nordjylland	0.5578	0.4925	0.6318	∨	0.0635	-9.1887	<0.0001

Table S12. Risk of bipolar disorder (DF30, DF31) by paternal age, maternal age and parental age difference at birth.

	exp(coef)	LCL	UCL	risk	SE	Z	Pr(Z)
P age group 1	1.4421	0.6243	3.3309	.	0.4271	0.8571	0.3914
P age group 2	1.0071	0.6621	1.5317	.	0.2139	0.0330	0.9737
P age group 3	0.9404	0.7219	1.2250	.	0.1349	-0.4554	0.6489
P age group 4	1.1567	0.8647	1.5474	.	0.1485	0.9808	0.3267
P age group 5	0.9383	0.5613	1.5683	.	0.2621	-0.2431	0.8079
P age group 6	0.9433	0.4160	2.1387	.	0.4177	-0.1399	0.8888
M age group 1	1.2398	0.8672	1.7726	.	0.1824	1.1787	0.2385
M age group 2	1.0195	0.7769	1.3378	.	0.1386	0.1393	0.8893
M age group 3	1.0677	0.8784	1.2978	.	0.0996	0.6580	0.5105
M age group 4	1.1785	0.9435	1.4720	.	0.1134	1.4478	0.1477
M age group 5	1.0314	0.7584	1.4027	.	0.1569	0.1970	0.8438
M age group 6	1.3074	0.8153	2.0964	.	0.2409	1.1125	0.2659
P-M group 1	1.7377	0.7482	4.0356	.	0.4299	1.2853	0.1987
P-M group 2	1.6319	1.0494	2.5379	∧	0.2253	2.1738	0.0297

P-M group 3	1.0212	0.8493	1.2280	.	0.0941	0.2231	0.8235
P-M group 4	1.0769	0.8606	1.3477	.	0.1144	0.6478	0.5171
P-M group 5	1.3954	0.9685	2.0105	.	0.1863	1.7880	0.0738
P-M group 6	1.3280	0.7090	2.4874	.	0.3202	0.8860	0.3756
maternal disorder	11.9153	8.0812	17.5684	∧	0.1981	12.5076	<0.0001
paternal disorder	8.4616	5.1404	13.9285	∧	0.2543	8.3980	<0.0001
gestation length	1.0096	0.9601	1.0617	.	0.0257	0.3741	0.7083
maternal bleeding	0.7804	0.5550	1.0974	.	0.1739	-1.4257	0.1539
fetal oxygen deprivation	0.5177	0.0726	3.6906	.	1.0021	-0.6570	0.5112
pregnancy oedema	0.4960	0.2052	1.1987	.	0.4502	-1.5575	0.1194
gestational diabetes	-	-	-	.	-	-	-
gestational hypertension	0.8777	0.5862	1.3142	.	0.2059	-0.6333	0.5265
APGAR5 score	0.8047	0.6518	0.9934	∨	0.1075	-2.0219	0.0432
birth weight (grams)	1.0000	0.9998	1.0002	.	0.0001	0.1413	0.8876
pre-existing hypertension	1.1652	0.1625	8.3567	.	1.0052	0.1521	0.8791
pre-existing diabetes	0.5408	0.0757	3.8652	.	1.0034	-0.6126	0.5402
previous abortion (induc.)	1.2310	1.0136	1.4951	∧	0.0992	2.0963	0.0361
previous abortion (spont.)	1.0089	0.8090	1.2583	.	0.1127	0.0790	0.9370
parity (2nd)	0.8442	0.6994	1.0190	.	0.0960	-1.7638	0.0778
parity (3rd)	0.8661	0.6595	1.1374	.	0.1390	-1.0340	0.3011
parity (4+)	0.6804	0.4291	1.0790	.	0.2353	-1.6369	0.1017
sex	1.7428	1.4837	2.0470	∧	0.0821	6.7663	<0.0001
education level	1.1502	1.0855	1.2188	∧	0.0295	4.7363	<0.0001
average income	0.9073	0.8774	0.9383	∨	0.0171	-5.6779	<0.0001
nationality	0.8955	0.5552	1.4444	.	0.2439	-0.4524	0.6510
birth year cohort	1.5052	1.3771	1.6452	∧	0.0454	9.0142	<0.0001
birth season	1.0274	1.0038	1.0515	∧	0.0118	2.2840	0.0224
region Sjælland	0.8862	0.6611	1.1878	.	0.1495	-0.8083	0.4189
region Syddanmark	1.2603	0.9959	1.5948	.	0.1201	1.9255	0.0542
region Midtjylland	1.6706	1.3427	2.0786	∧	0.1115	4.6027	<0.0001
region Nordjylland	1.1077	0.8234	1.4901	.	0.1513	0.6757	0.4992

Table S13. Risk of major depression (DF32, DF33) by paternal age, maternal age and parental age difference at birth.

	exp(coef)	LCL	UCL	risk	SE	Z	Pr(Z)
P age group 1	1.0674	0.8896	1.2806	.	0.0929	0.7014	0.4830
P age group 2	1.1015	1.0128	1.1981	∧	0.0429	2.2559	0.0241
P age group 3	1.0245	0.9707	1.0812	.	0.0275	0.8791	0.3794
P age group 4	1.0041	0.9438	1.0681	.	0.0316	0.1284	0.8978
P age group 5	0.9639	0.8674	1.0712	.	0.0538	-0.6828	0.4947
P age group 6	0.9226	0.7793	1.0922	.	0.0861	-0.9360	0.3493
M age group 1	1.2520	1.1392	1.3759	∧	0.0482	4.6648	<0.0001
M age group 2	1.1385	1.0615	1.2211	∧	0.0357	3.6298	0.0003
M age group 3	1.0074	0.9577	1.0598	.	0.0258	0.2872	0.7739
M age group 4	1.0531	0.9927	1.1173	.	0.0302	1.7167	0.0860
M age group 5	1.0551	0.9740	1.1431	.	0.0408	1.3144	0.1887

M age group 6	1.0078	0.8842	1.1486	.	0.0667	0.1157	0.9079
P-M group 1	1.1706	0.9162	1.4957	.	0.1250	1.2598	0.2078
P-M group 2	1.1596	1.0226	1.3150	∧	0.0642	2.3082	0.0210
P-M group 3	0.9906	0.9436	1.0399	.	0.0248	-0.3823	0.7022
P-M group 4	1.0769	1.0163	1.1411	∧	0.0295	2.5074	0.0122
P-M group 5	1.1597	1.0507	1.2801	∧	0.0504	2.9417	0.0033
P-M group 6	1.2034	1.0206	1.4190	∧	0.0841	2.2026	0.0276
maternal disorder	2.4213	2.2790	2.5725	∧	0.0309	28.6154	<0.0001
paternal disorder	2.0769	1.9234	2.2427	∧	0.0392	18.6538	<0.0001
gestation length	0.9914	0.9816	1.0013	.	0.0051	-1.7027	0.0886
maternal bleeding	1.1827	1.1166	1.2528	∧	0.0294	5.7149	<0.0001
fetal oxygen deprivation	0.9886	0.7090	1.3785	.	0.1696	-0.0674	0.9463
pregnancy oedema	0.9645	0.8406	1.1067	.	0.0702	-0.5146	0.6068
gestational diabetes	-	-	-	.	-	-	-
gestational hypertension	1.0415	0.9646	1.1245	.	0.0391	1.0385	0.2990
APGAR5 score	0.9513	0.9068	0.9979	∨	0.0244	-2.0461	0.0407
birth weight (grams)	1.0000	0.9999	1.0000	∨	0.0000	-2.3816	0.0172
pre-existing hypertension	1.5230	1.0804	2.1468	∧	0.1752	2.4013	0.0163
pre-existing diabetes	1.0271	0.7810	1.3506	.	0.1397	0.1911	0.8485
previous abortion (induc.)	1.1382	1.0941	1.1841	∧	0.0202	6.4239	<0.0001
previous abortion (spont.)	1.0783	1.0321	1.1265	∧	0.0223	3.3765	0.0007
parity (2nd)	1.0906	1.0495	1.1333	∧	0.0196	4.4267	<0.0001
parity (3rd)	1.1683	1.1044	1.2358	∧	0.0287	5.4231	<0.0001
parity (4+)	1.1902	1.0898	1.2998	∧	0.0450	3.8720	0.0001
sex	2.4426	2.3594	2.5288	∧	0.0177	50.4725	<0.0001
education level	1.0020	0.9902	1.0140	.	0.0061	0.3381	0.7353
average income	0.9199	0.9135	0.9264	∨	0.0036	-23.4838	<0.0001
nationality	0.3354	0.2980	0.3774	∨	0.0603	-18.1290	<0.0001
birth year cohort	1.4157	1.3941	1.4377	∧	0.0079	44.1814	<0.0001
birth season	1.0104	1.0056	1.0152	∧	0.0024	4.2886	<0.0001
region Sjælland	0.7835	0.7424	0.8268	∨	0.0275	-8.8864	<0.0001
region Syddanmark	0.9547	0.9130	0.9984	∨	0.0228	-2.0320	0.0422
region Midtjylland	1.0245	0.9807	1.0703	.	0.0223	1.0849	0.2780
region Nordjylland	0.5432	0.5078	0.5810	∨	0.0344	-17.7592	<0.0001

Table S14. Risk of schizophrenia-schizotypal-delusional disorders (DF20-29) by paternal age, maternal age and parental age difference at birth.

	exp(coef)	LCL	UCL	risk	SE	Z	Pr(Z)
P age group 1	1.0251	0.7962	1.3198	.	0.1289	0.1923	0.8475
P age group 2	0.9771	0.8598	1.1103	.	0.0652	-0.3556	0.7221
P age group 3	0.9634	0.8865	1.0469	.	0.0424	-0.8792	0.3793
P age group 4	1.0059	0.9144	1.1067	.	0.0487	0.1218	0.9031
P age group 5	1.1482	0.9834	1.3406	.	0.0790	1.7485	0.0804
P age group 6	1.1043	0.8685	1.4042	.	0.1226	0.8097	0.4181
M age group 1	1.5546	1.2950	1.8662	∧	0.0932	4.7335	<0.0001
M age group 2	1.1679	1.0153	1.3435	∧	0.0715	2.1720	0.0299

Supporting Information: Tables

M age group 3	1.0212	0.9216	1.1316	.	0.0524	0.4002	0.6890
M age group 4	0.9672	0.8562	1.0926	.	0.0622	-0.5362	0.5918
M age group 5	0.9345	0.7937	1.1004	.	0.0833	-0.8125	0.4165
M age group 6	0.8835	0.6789	1.1497	.	0.1344	-0.9216	0.3567
P-M group 1	1.5508	1.1117	2.1634	∧	0.1699	2.5832	0.0098
P-M group 2	1.3008	1.0787	1.5686	∧	0.0955	2.7534	0.0059
P-M group 3	1.0460	0.9702	1.1279	.	0.0384	1.1717	0.2413
P-M group 4	1.0612	0.9719	1.1587	.	0.0448	1.3251	0.1851
P-M group 5	1.1594	1.0036	1.3393	∧	0.0736	2.0092	0.0445
P-M group 6	1.3567	1.0809	1.7029	∧	0.1160	2.6305	0.0085
maternal disorder	3.4002	3.0147	3.8351	∧	0.0614	19.9312	<0.0001
paternal disorder	2.4531	2.1264	2.8301	∧	0.0729	12.3038	<0.0001
gestation length	0.9915	0.9768	1.0064	.	0.0076	-1.1235	0.2612
maternal bleeding	1.2043	1.1050	1.3125	∧	0.0439	4.2341	<0.0001
fetal oxygen deprivation	1.0150	0.5883	1.7512	.	0.2783	0.0535	0.9573
pregnancy oedema	1.0504	0.8603	1.2824	.	0.1018	0.4827	0.6293
gestational diabetes	-	-	-	.	-	-	-
gestational hypertension	1.0618	0.9450	1.1931	.	0.0595	1.0089	0.3130
APGAR5 score	0.9866	0.9162	1.0623	.	0.0377	-0.3585	0.7199
birth weight (grams)	0.9999	0.9998	1.0000	∨	0.0000	-3.7324	0.0002
pre-existing hypertension	0.7605	0.3617	1.5991	.	0.3792	-0.7221	0.4703
pre-existing diabetes	1.2670	0.8602	1.8663	.	0.1976	1.1977	0.2310
previous abortion (induc.)	1.2237	1.1540	1.2976	∧	0.0299	6.7499	<0.0001
previous abortion (spont.)	1.0055	0.9388	1.0768	.	0.0350	0.1559	0.8761
parity (2nd)	1.0345	0.9750	1.0977	.	0.0302	1.1217	0.2620
parity (3rd)	1.1510	1.0568	1.2537	∧	0.0436	3.2274	0.0012
parity (4+)	1.1295	0.9925	1.2855	.	0.0660	1.8465	0.0648
sex	0.8403	0.7998	0.8829	∨	0.0252	-6.8938	<0.0001
education level	1.0206	1.0025	1.0389	∧	0.0091	2.2402	0.0251
average income	0.8593	0.8503	0.8683	∨	0.0053	-28.3605	<0.0001
nationality	0.6195	0.5485	0.6996	∨	0.0620	-7.7187	<0.0001
birth year cohort	1.2275	1.2005	1.2552	∧	0.0114	18.0228	<0.0001
birth season	1.0061	0.9989	1.0134	.	0.0037	1.6547	0.0980
region Sjælland	0.6502	0.6013	0.7032	∨	0.0399	-10.7799	<0.0001
region Syddanmark	0.7203	0.6742	0.7696	∨	0.0338	-9.7131	<0.0001
region Midtjylland	0.5930	0.5529	0.6361	∨	0.0358	-14.6131	<0.0001
region Nordjylland	0.4905	0.4455	0.5400	∨	0.0491	-14.5217	<0.0001

Table S15: Correlation between maternal age and birth-related traits for offspring

offspring trait	gender**	*r*	95% CI	*t*-value	*P*-value
**birth weight***	**m,f**	0.112	0.111-0.113	150.71	<0.001
	**m**	0.113	0.111-0.115	108.86	<0.001
	**f**	0.113	0.111-0.116	106.56	<0.001
**birth length***	**m,f**	0.092	0.091-0.094	123.96	<0.001
	**m**	0.092	0.090-0.094	88.5	<0.001
	**f**	0.096	0.094-0.098	89.79	<0.001
**birth bmi***	**m,f**	0.074	0.073-0.076	98.43	<0.001
	**m**	0.075	0.073-0.077	70.98	<0.001
	**f**	0.074	0.072-0.076	68.29	<0.001
**head circumference***	**m,f**	0.076	0.074-0.079	65.23	<0.001
	**m**	0.077	0.074-0.080	47.16	<0.001
	**f**	0.075	0.075-0.082	46.92	<0.001
**abdominal circumference***	**m,f**	0.067	0.067-0.072	58.68	<0.001
	**m**	0.066	0.066-0.073	41.76	<0.001
	**f**	0.067	0.067-0.074	41.34	<0.001
**APGAR5**	**m,f**	0.0015	0.00008-0.00303	2.07	0.037
	**m**	0.0016	-0.0003-0.0037	1.6	0.108
	**f**	0.0014	-0.0006-0.0035	1.33	0.183
**placenta weight**	**m,f**	0.061	0.058-0.063	49.45	<0.001
	**m**	0.058	0.055-0.061	33.83	<0.001
	**f**	0.064	0.060-0.067	36.27	<0.001

Footnotes:
Estimates are Pearson correlation
* adjusted for gestation length
** offspring gender: m,f=male and female offspring included; m=only male offspring; f=only female offspring
