# Supplementary material for: Opposite differential risks for autism and schizophrenia based on maternal age, paternal age, and parental age differences
Source: Evol Med Public Health. 2016 Aug 16;2016(1):286–98. doi: 10.1093/emph/eow023 (PMC5026125; doi:10.1093/emph/eow023)
Supplement: Supplementary Data [file eow023_Supp.zip › eow023-suppl_data/Supplementary_Methods_Results.pdf]

Supplementary Methods & Results

Sources and validation of mental illness. Offspring were classified as having a mental illness if they had been admitted as an inpatient or had been in outpatient care. In Denmark, the International Classification of Diseases, 8th revision (ICD-8) was used from 1969 to 1993, and the 10th revision (ICD-10) from 1994 onwards (Table S3). Up to 30 years of follow-up was available for each offspring up until 2009. Individuals suspected of having a mental illness are referred by general practitioners or school psychologists to a psychiatric clinic where they are diagnosed and treated by psychiatrists. In our sample of cases, we reduced the potential for early misdiagnoses (or differences in the ICD diagnostic criteria over time) by tracking individual's psychiatric history and taking their most recent diagnosis as the actual diagnosis and the first for censoring time in the Cox regression model. Psychiatric history for parents of the offspring sampled was also scanned for the same diagnoses and binary presence/absence markers were included in analyses to help account for the known strong heritable component for autistic and schizophrenic disorders. Cox regression was run for each psychiatric disorder group with any offspring removed a-priori who had a non-target psychological disorder. This reduced any confounding effect on the comparisons between offspring diagnosed with a focal disorder and the controls with no history of mental illness. To remove confounding of drug use on risk of psychiatric disorders, individuals (parents or offspring) were also excluded if they were ever recorded with a psychiatric disorder due to drug or alcohol use (ICD-10 codes F10-F19; ICD-8 codes 291.09, 291.19, 291.29, 291.39, 291.99, 303.09, 303.19, 303.20, 303.28, 303.29, 303.90, 303.91, 303.99, 304.09, 304.19, 304.29, 304.39, 304.49, 304.59, 304.69, 304.79, 304.89, 304.99).

Risk patterns for covariates (continued). The effects of potentially confounding factors are given in Supplementary Tables S5-S14. Across the narrow and broad spectrum categories of mental diseases that we analyzed while taking 22 covariates into account an average of 51% were significant at the $P<0.001$ level. This confirms that covariates are important and that not having them available may affect the conclusions of large scale studies on mental disease. Some of these effects are worth mentioning. First, pregnancy complications such as maternal bleeding and a history of previous abortions increases risks for both types of mental disease, but the effect on schizophrenic disorders is stronger than the effect on autistic disorders. Second, education level has no effect on either type of mental disease, but average income decreases both risk types, suggesting that, after parental age factors have been taken into account, factors of economic prosperity matter even in a country like Denmark where income differences are among the lowest in the world. Third, non-Danish nationals have systematically reduced risks for all types of mental disease, similar to Danes from rural areas compared to urban areas. Whether this effect is real or due to some degree of under diagnosis remains to be established, because starting families at younger parental age cannot explain this effect. Fourth, birth season has a consistent effect on al types of mental diseases, indicating that infants born later in the calendar year have increased risks of being diagnosed with mental disorders. Lastly, for some autistic disorder groups, risk was lower in later born offspring and for some schizophrenic disorder groups higher in later born offspring. This may appear to run counter to expectations based on observed risk depending

on maternal and paternal age. However we should be cautious when comparing these two effects as parity does not capture the same scale of temporal variation as parental age due to differing interbirth intervals, and may in fact be capturing some other age-related environmental effects (more likely than capturing an additional biological effect) related to parental age per se. This would thus require further investigation. Moreover, the effect of parity was not consistent across all autistic (4 of 5) and schizophrenic (2 of 5) disorder groups.
